# The Good, the Bad, and the Fungus: Insights into the Relationship Between Plants, Fungi, and Oomycetes in Hydroponics

**DOI:** 10.3390/biology13121014

**Published:** 2024-12-04

**Authors:** Grace C. S. Laevens, William C. Dolson, Michelle M. Drapeau, Soufiane Telhig, Sarah E. Ruffell, Danielle M. Rose, Bernard R. Glick, Ashley A. Stegelmeier

**Affiliations:** 1 Department of Biology, University of Waterloo, 200 University Avenue West, Waterloo, ON N2L 3G1, Canadawdolson@uwaterloo.ca (W.C.D.); mdrapeau@uwaterloo.ca (M.M.D.);; 2 Ceragen Inc., 151 Charles St W, Suite 199, Kitchener, ON N2G 1H6, Canada; sofiane.telhig@ceragengrow.com (S.T.);

**Keywords:** hydroponics, fungi, fungal microbiome, food production, oomycetes, controlled environment agriculture

## Abstract

Hydroponic systems are examples of controlled environment agriculture (CEA) and present a promising alternative to traditional farming methods by increasing productivity, profitability, and sustainability. In hydroponic systems, crops are grown in the absence of soil and thus lack the native soil microbial community. This review focuses on fungi and oomycetes, both beneficial and pathogenic, that can colonize crops and persist in hydroponic systems. The symptomatology and mechanisms of pathogenesis for *Botrytis*, *Colletotrichum*, *Fulvia*, *Fusarium*, *Phytophthora*, *Pythium*, and *Sclerotinia* are explored for phytopathogenic fungi that target floral organs, leaves, roots, and vasculature of economically important hydroponic crops. Additionally, this review thoroughly explores the use of plant growth-promoting fungi (PGPF) to combat phytopathogens and increase hydroponic crop productivity; details of PGP strategies and mechanisms are discussed. The benefits of *Aspergillus*, *Penicillium*, *Taloromyces*, and *Trichoderma* to hydroponics systems are explored in detail. The culmination of these areas of research serves to improve the current understanding of the role of beneficial and pathogenic fungi, specifically in the hydroponic microbiome.

## Simple Summary

A range of fruits and vegetables including tomatoes, cucumbers, peppers, and lettuce are grown in hydroponic systems. These systems do not contain soil and instead use a circulating nutrient solution to grow the plants. Removing soil from the growing systems changes the microscopic organisms that are present at the root of the plants. Fungi and oomycetes, a fungal-like organism, are both present in these systems. Some of these organisms promote plant growth, while others cause disease and crop loss. This paper reviews current literature on seven organisms that cause disease in plants, as well as four beneficial organisms. We explain the strategies beneficial fungi use to help plants grow, including direct killing of pathogens, strengthening the plant’s immune system, and enabling the plant to gain more nutrients from the environment. Additionally, we explain how phytopathogens spread throughout hydroponic systems and destroy plant tissue. The aim of this review is to provide readers with management strategies to reduce harmful plant diseases in hydroponic operations, as well as beneficial options to increase crop yields. Both scientists and growers can benefit from a complete understanding of the interactions between plants, fungi, and oomycetes.

## 1. Introduction

According to the United Nations’ most recent projection, the global population is expected to increase to 9.8 billion by 2050 [1]. Population increases directly result in greater global food demand. To meet current 2050 demand projections, agricultural productivity must increase annually by an estimated 0.5–1% [2]. Unfortunately, both the quantity and quality of farmland have decreased dramatically in the last several decades. For example, arable farmland in Canada decreased by 8% between 2001 and 2021 as cropland was developed for urban centers [3]. Additionally, the quality of more than half of all agricultural land worldwide has been significantly degraded by erosion, salinization, acidification, and contamination [4]. Therefore, novel strategies that increase sustainable production of quality crops are required to feed nearly 10 billion people using less arable land than currently exists.

The use of controlled environment agriculture (CEA) presents one viable solution to this problem. Also referred to as indoor farming, CEA is the growth of crops under partially or completely controlled conditions [5]. A high tunnel is a 3–4 m tall simple framed hoop structure that is coated in polyethylene, fabric, or plastic to extend the growing season. CEA systems range from “low-tech” options, such as high tunnels, to “high-tech” greenhouses equipped with lighting, temperature, and humidity control [5]. Specifically, hydroponic systems are CEA systems that use water-based nutrient solutions and inert substrates instead of soil [6].

Hydroponics are more productive and sustainable than traditional farming, with significantly higher yields and dramatically lower fertilizer [7] and water demands [8] due to recirculating water [6]. Vertical farms in particular are able to achieve significantly higher yields per unit area by stacking systems to take advantage of vertical space [9]. Many growers are choosing to adopt CEA practices, with the hydroponic market size expected to grow at a compound annual growth rate of 12.4% from 2024 to 2030 [10]. 

**Figure 1:**
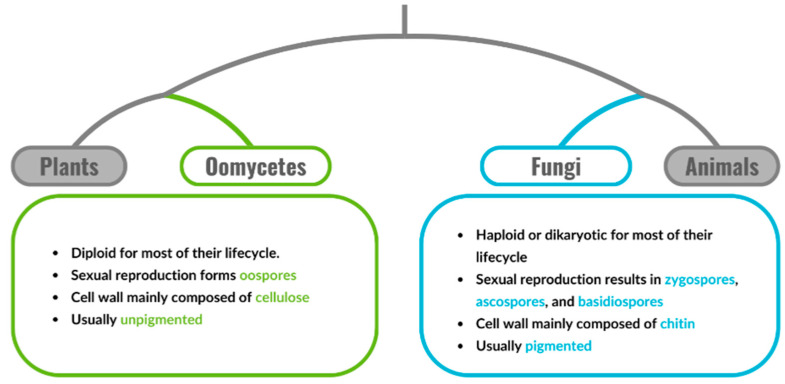
Comparing and contrasting characteristics of oomycetes and true fungi. Oomycetes (green) and true fungi (blue) are highly similar, but differ in a few key characteristics: pigmentation, cell wall composition, dominant life cycle ploidy, and spore production.

The roots of hydroponically grown crops are colonized by a wide variety of microorganisms, including bacteria and eukaryotic microbes such as fungi and oomycetes [11]. Fungi are a diverse group of eukaryotic organisms characterized by filamentous vegetative hyphal networks. Oomycetes were previously taxonomically classified as fungi. However, they are now classified as part of the phylum *Stramenopiles* due to improved resolution from molecular studies. True fungi and oomycetes inhabit comparable ecological niches; however, they differ in their major cell wall components, sexual reproduction strategies, and vegetative nuclear states (Figure 1) [12]. Like bacteria, the interaction between eukaryotic microbes and plants may be beneficial, harmful, or neutral [13].

Hydroponic systems were initially developed to reduce water consumption, soil degradation, and as a strategy to circumvent soilborne phytopathogens [14]. It was hypothesized that growing the plants in soilless cultures would be an effective tool in curbing soil-based phytopathogen infections. However, the practice of recirculating water and the uniformity of nutrient distribution amongst plants can result in infection from many fungi and oomycetes [14]. Some of these phytopathogens, like *Fusarium oxysporum*, are considered opportunistic pathogens, usually existing in soil around healthy plants but unable to infect them. These organisms may display different patterns of infection in a controlled, indoor soilless environment [15]. A complete understanding of the microbiome has the potential to increase crop yields via controlling harmful fungal pathogens or using some fungi to promote plant growth. This review aims to summarize the role of the fungal biome in hydroponics, both in disease pathology and plant growth promotion. 

## 2. Fungal Pathogens

Historically, fungal infections have been an ever-present and at times destructive occurrence in agriculture, extending to long-lasting societal implications. Indeed, the severity of fungal infections has been recorded on many occasions, including the devastation of coffee plantations in Sri Lanka and the Irish potato famine in the 19th century [16,17]. Fungi are highly diverse and prevalent, potentially infecting a wide range of key crops. In addition, pathogenic fungi also constitute an alarming threat to hydroponic cultures [18,19]. Hydroponic systems optimize plant growth and health in part with a carefully monitored nutrient solution system. However, this optimized homogeneity may lead to an increased risk of phytopathogen propagation [20]. 

**Figure 2:**
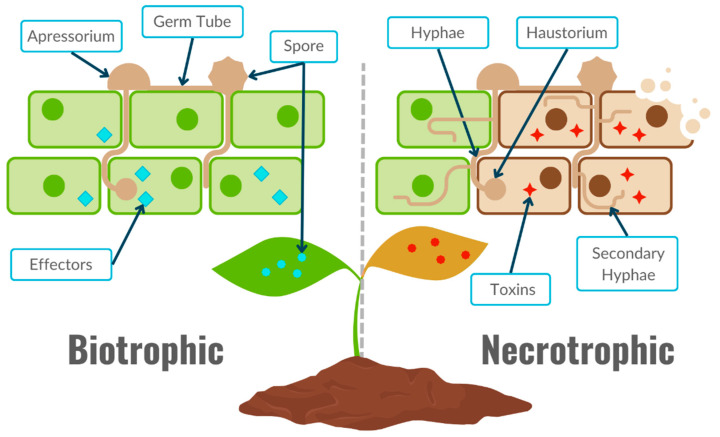
Comparison of biotrophic and necrotrophic phytopathogen behaviors. Fungal and oomycete interactions in plants are categorized based on their effect on the plant tissue. Biotrophic interactions tend to preserve tissue health, where the parasite (oomycete or fungus) siphons off nutrients from healthy tissue. Whereas necrotrophic parasites cause host cell death, freeing nutrients and allowing saprophytic feeding.

Plant pathology of fungi and oomycetes was historically divided into three lifestyles: biotrophic, hemibiotrophic, and necrotrophic (Figure 2) [18]. Biotrophic phytopathogens are microbes that infect and syphon off nutrients from the host tissue, without leading to its death. In contrast, necrotrophic phytopathogens gain access to nutrients by targeting and killing the host tissue to feed saprophytically on the resulting necrotic tissue. Hemibiotrophs are phytopathogens that exhibit an initial biotrophic phase, where the phytopathogen establishes itself and makes its way to the desired organ. After which a necrotrophic phase takes place [18]. Many phytopathogens, including both fungi and oomycetes, exhibit biotrophic and necrotrophic behavior, which speaks to the complexity of interactions and the inaccuracy of the previous lifestyle classification. Pathogenic fungi can cause visible signs of disease on the host during classical biotrophic pathways [19]. Biotrophs secrete effectors that modulate the plant’s immune system response and inhibit apoptosis to safely extract nutrients from healthy tissue while preserving its structural integrity. This strategy results in mild but economically deleterious symptoms on the host plant, such as mold, powdery mildew, and rusts [21,22]. Mold and powdery mildew generally grow on the aerial organs of plants and constitute the transmission vectors of the phytopathogen as ready-to-germinate propagules [19]. In contrast, rusts cause acute inhibition of the natural immune response, and apoptosis can lead to uncontrolled growth on the plant [21,22]. The latter symptom usually affects crops such as wheat, soybean, maize, and coffee, which are rarely grown in hydroponics systems. Hence, biotrophic phytopathogens that exhibit mold and powdery mildew are more common in hydroponics settings; however, their molecular mechanisms have been investigated less than necrotrophic organisms. 

Conversely, necrotrophic phytopathogens possess a range of virulence factors, as their lifecycle induces necrosis to feed on dead tissue. Strategies include secreting cell wall degrading enzymes (CWDEs) or oxalic acid to suppress the host plant’s oxidative burst [23,24]. Various approaches result in a range of symptoms on the plant, including root rot, crown rot, vascular wilt, fruit rot, or fruit blight [25]. The following section provides a general overview of the pathology of the most relevant fungal and oomycetes phytopathogens found in hydroponic systems. 

## 3. Phytopathogens Targeting Floral and Leaf Organs

### 3.1. Botrytis cinerea

Botrytis is a genus of true fungi that contains 32 species [26], with most of them classified as true necrotrophic pathogens except for *Botrytis deweyae*, which grows almost asymptomatically within *Hemerocallis* daylilies as an endophyte [27]. One of the most notorious fungal phytopathogens is *Botrytis cinerea*. *Botrytis* spp. usually target specific plant species [28], but *B. cinerea* is a generalist, causing gray mold in over 200 plant species. In hydroponics, it affects multiple crops such as tomato, bell-pepper, strawberry, cucumber, and eggplant [29,30]. The infection cycle starts when fungal spores encounter floral or leaf organs of the host plant, appearing as light tan or gray spots. Contact with the appropriate organ and at high, >80%, humidity induces germination of the fungal spores [19], which propagate and seek entry into host plants through either damaged tissue or stomata [28]. The stigmatic fluid provides nutrients for the fungal germination phase, supporting the growth of hyphae invading the host tissue. In the beginning of this infection cycle, *B. cinerea* coexists with host cells as a biotroph [28] with little to no symptoms. However, the ripening of the plant tissue during the fruiting stage changes the composition of the volatile organic compounds, sugars, and nitrogen in the host tissue. For instance, in both tomato and strawberry fruits, the volatile organic compound (E)-2-hexenal upregulates sulfate assimilation in *B. cinerea* spores and hyphae, which contributes to disease severity [31]. Indeed, ripening can act as a switch, transitioning the fungus from a biotrophic to a necrotrophic phase [32]. During this stage, the fungus secretes necrotrophic virulence factors such as oxalic acid and cell wall-degrading enzymes, leading to host cell death. Cell wall degradation releases nutrients that can be consumed saprophytically, as demonstrated in Figure 2 [33]. In contrast, oxalic acid is believed to be necessary for *B. cinerea* virulence through its reduction in the pH of host cells [34,35]. This correlates with [36], who observed an increase in gray mold resistance in hydroponically grown paprika plants in a higher pH nutrient solution by changing the source of inorganic calcium being added to the nutrient solution. They have observed the highest increases of disease resistance in the plants being supplied by the nutrient solutions with the highest pH (8.2–10 in comparison to 6.6). Conversely, silicon complementation to nutrient solution in hydroponic systems contributed to a transient decrease in *B. cinerea* disease severity in tomatoes, lettuce, and cucumber plants [37]. Silicon comprises 0.1–10% of dry plant biomass and is structurally incorporated into the epidermis and cuticle. Supplementation with this micronutrient resulted in increases to cuticle thickness ranging from 10.0–58.0%. Interestingly, the plant with the most significant disease protection, lettuce, also exhibited the highest increase in cuticle thickness (58.0%) when treated with silicon additives. Thus, ensuring nutrient solutions contain adequate quantities of silicon for optimal cuticle thickness is an inexpensive strategy for reducing Botrytis disease severity. The use of silicon in agriculture to improve plant health has been reviewed extensively [38]. Reviewers determined ~34% of silicon studies were conducted in hydroponic systems. Silicon supplementation was found to positively impact toxicity (30–38%), drought stress (21–29%), disease resistance (17–21%), and growth promotion (12–17%). Of note, silicon supplementation was more beneficial in reducing diseases in hydroponics than soil-based agriculture.

Once a *B. cinerea* infection is established and allowed to progress, the fruit tissue of the host starts to soften, turn brown, and become leathery. Furthermore, the new intake of nutrients from the necrotic fruit supports the sexual reproduction of the next generation of the fungus and gray mold formations [28]. Such transitions are finely tuned through a complex transcriptomic process affecting both the host and the pathogen. For instance, a recent study by Chen and colleagues observed that the transition of plant defense mechanisms in *Arabidopsis thaliana* was suppressed in a *B. cinerea* mutant lacking the transcriptomic factor BcSpd1 [39]. BcSpd1 modulates other pathogenicity factors controlling invasion through infection cushions and appressoria, which are fungal structures used for host cell wall penetration [28]. In addition, a mutant of BcSpd1 was not able to produce any sclerotia and exhibited an overproduction of melanin, an important fungal pigment. Furthermore, it was observed that BcSpd1 can reduce the expression of antifungal flavonoids in infected ginseng plants. These findings suggest that the reproductive cycle of the fungus is also modulated by this factor. Sclerotia is the main reproductive propagule of fungi [40], and melanin is a natural pigment produced by fungi, allowing them to survive stringent environmental conditions [41].

As a generalist capable of infecting many agricultural crops, *B. cinerea* is in frequent contact with fungicides commonly used in agricultural fields and greenhouses [42]. *B. cinerea*’s resistance to fungicides is mediated through the major facilitator superfamily of efflux pumps [43]. These active transporters can reduce the intracellular concentration of a broad range of fungicides by ejecting them from the fungal cytoplasm [44]. Indeed, once the disease has progressed, the possibility of effective management with chemicals is very limited; hence, it is important to implement other forms of disease control. 

Gray mold generally spreads in hydroponics when substandard sanitation protocols are implemented. Controlling *B. cinerea* commences with implementing periodic sanitation of the greenhouse between growth cycles, especially when the facility is empty [45]. Furthermore, as the fungus targets plant leaves for infection and transmission, it is recommended to control the microenvironment of the canopy. First, as the fungal spores can be transmitted through moisture and germinate in high humidity levels (80%), it is important to keep the leaf surfaces as dry as possible. This can be achieved with adequate spacing between the plants to increase ventilation, reduce relative humidity, and control the temperature inside the greenhouse [29]. Second, overhead watering of plants should be avoided. Third, there are few resistant cultivars to *B. cinerea* that have the capacity of developing fungicide resistance [43]. Overlaying multiple disease control methods and alternating sanitation chemicals and fungicides is highly recommended [29]. Concomitantly, [46] have observed a marked reduction (68%) of *B. cinerea* disease symptoms in hydroponically grown lettuce leaves using Corn Steep Liquor (CSL). Further molecular analysis revealed an increase in *Ls-ACS1* expression in the CSL-grown plants in comparison to the lettuce plants grown in a standard inorganic nutrient solution, which is the gene encoding ACC synthase in lettuce plants. Similar effects were also observed in cucumber plants grown in a CSL solution, whereby they exhibited a higher activation of their Jasmonic acid/ethylene response. This suggests that it is possible to induce lettuce and cucumber plant systemic resistance (ISR) with organic nutrient solution as a means of controlling *B. cinerea* disease incidence in hydroponics.

### 3.2. Fulvia fulva

Previously called *Cladosporium fulvum* and *Passalora fulva* [47], *Fulvia fulva* is a true fungus and is the causative agent of leaf mold. It is primarily a disease of greenhouse-grown tomatoes and has been a model for studying plant-fungal molecular interactions [48]. According to the recent classification proposed by [49], *F. fulvia* is closer to a hemibiotroph than a true biotroph given that its genomes contain sequences coding for glycoside hydrolases, which are associated with cell host death and are regarded as damage-associated molecular patterns, recognizable by the host immune system [50]. The infection cycle starts when fungal spores germinate at high humidity (over 85%) and start growing hyphae in random directions over the leaf surface [47]. Entry into the host happens when the hyphae encounter an open stoma, at which point growth of the hyphae is limited to the apoplastic space between mesophyll cells [47]. This growth appears to be directed towards the vascular system, suggesting some form of chemotaxis by the gradient of nutrients [51]. At this stage, minimal symptoms are visible from the host, aside from the occasional callose deposition on the mesophyll cell walls. This lack of host tissue disruption is responsible for the long latent abiotrophic phase of *F. fulvia*. The host range of this fungus is restricted to the genus *Lycopersicon*, with many tomato crops and cultivars exhibiting innate resistance to the fungus. The plant can detect the entry of the fungus and induce a hypersensitive response (HR) whereby the tissue surrounding the invading hyphae collapses on itself, effectively containing the fungus in a physically enclosed space [52]. It is this HR that made *F. fulvia* a model for host–pathogen molecular interactions, which lead to the identification of several virulence genes dubbed *Avr* (“avirulent”). The successful recognition of pathogen associated molecular patterns (PAMPs) and/or fungal effectors by the host plant can lead to effective fungal containment [53]. However, if successful infection is established by *Fulvia*, the hyphae continue growing along the apoplast of the vascular system until the formation of aerial mycelium protrudes from open stomata, containing the conidia of the next generation [47].

In hydroponic systems, *F. fulvia* is a global causative agent of leaf mold, also called “brown leaf mold” in tomatoes [54,55,56], resulting in defoliation of the plants and fungal growth on the underside of the leaves. In the beginning of the disease, the leaves exhibit olive-green to grayish purple fungal spots that later turn yellowish brown. Resistance to *F. fulvia* infections is governed by the *Cladosporium fulvum* resistance genes (*Cf*). They encode cell surface receptor-like proteins (RLPs), which recognize *F. fulvia* effectors to activate plant immune responses [57,58]. In many commercial tomato cultivars, resistance to this fungus is controlled by the *Cf*-9 locus, which encodes five paralogous RLPs, capable of identifying *F. fulvia* effectors and conferring resistance to the plants [53]. In a very recent study, 190 geographically diverse *F. fulvia* strains capable of breaking *Cf*-9 resistance exhibited significant mutations on their *Avr9B* gene accompanied by *Avr9* deletion [59]. Researchers recorded, in a greenhouse inoculation experiment, the presence of *F. fulvia* strains presenting resistance to multiple Succinate Dehydrogenase Inhibitors (SDHI) fungicides [60]. This apparent resistance to SDHIs was not correlated with any mutations on the *Sdh B* gene, which codes for the target of these fungicides, suggesting the involvement of cross-resistance mechanisms. To effectively control the disease, it is important to control the microenvironment of the canopy and keep it as dry as possible, especially before nightfall [29]. To achieve this, ventilation and spacing between plant canopies are important.

### 3.3. Colletotrichum

*Colletotrichum* spp. are true fungi that are phytopathogens of concern due to (i) their capacity to virtually infect every major cultivated crop [61], (ii) the lack of robust identification tools challenges of their taxonomical classification [62], and (iii) their capacity to induce post-harvest complications [63]. In hydroponics, *Colletotrichum* infections cause fruit rot symptoms in strawberries [64]. Their classification has been an ongoing subject of debate due to the lack of morphological characteristics of this genus [65]. Currently, the different fungal species are grouped in racial complexes, dependent on their morphology and genetic determinants. *Colletotrichum* classification remains an ongoing area of study, with new complexes continually being added [65]. Some complexes of *Colletotrichum* are known to co-exist as endophytes or biotrophs with specific plants. For example, the *Gloeosporioides* complex, while pathogenic in strawberry plants, is an endophyte of tropical grass species [66]. *Colletotrichum* spp. are ubiquitous worldwide [67,68,69] and can remain quiescent in seemingly “resistant” plants, later using them as a transmission vector to susceptible plants. *Colletotrichum* spp. are the main causative agent of anthracnose in strawberries grown hydroponically [61], but also infect other hydroponic crops such as pepper, tomato, cucumber, and spinach [63]. The name anthracnose, derived from the Greek words denoting “coal disease”, is used to describe dark necrotic lesions forming on the fruit of infected plants. Three *Colletotrichum* complexes are responsible for anthracnose in hydroponically grown strawberries: *C. acutatum*, *C. fragariae*, and *C. gloeosporioides* [61]. 

The general life cycle of these fungi is biphasic, with an initial biotrophic phase preceding a destructive necrotrophic phase that causes visible symptoms on the fruit (Figure 2) [61]. Based on the *Colletotrichum* racial complex, certain host infection sites are preferred by the pathogen [70]. Studies have shown that *C. gloeosporioides* and *C. acutatum* differ in their target organs [70]. For instance, *C. gloeosporioides* is most often associated with crown rot infections in strawberries, whereas *C. acutatum* is the main fruit rot pathogen [71]. All *Colletotrichum* spp. predominantly damage the strawberry fruit and crown; however, other symptoms such as leaf spotting and wilting can also be observed [72]. The fungus can persist asymptomatically on leaves and stems, which creates vector sources. In hydroponically grown strawberries, young plants are transferred from propagators to greenhouses, enabling *Colletotrichum* spp. to spread between facilities [73]. Concomitantly, these fungi can persist or “overwinter” in the fruit, resulting in postharvest spread [74,75]. Traditionally, *Colletotrichum* management is done through synthetic fungicides such as mancozeb, carbendazim, prochloraz, and Tecto 6 [76,77]. However, given the increase in fungicide resistance emergence [78] and the inherent cytotoxicity of these synthetic compounds [79], other control methods are being developed, such as exploiting antagonistic microbial interactions [80]. Interestingly, [81] observed that hydroponically grown bell pepper plants on a silicon-enriched inert medium (rice hull) significantly reduced disease score, up to 83%. Further, this was accompanied by an increase in cuticle thickness and cell wall-bound phenolic compounds of the fruits.

Asymptomatic plant tissue and infested soil from nurseries is the most common source of *Colletotrichum* inoculums in strawberry growing operations. Crown infection often occurs in nursery plants and only develops symptoms after planting in the greenhouse, making disease management difficult. To effectively address this problem, growers should monitor newly received plants for pathogens before their introduction into the main growing operation. Should early symptoms appear on the plants, they must be culled and removed from the greenhouse to reduce inoculum levels. Otherwise, anthracnose fruit rot can be controlled partly with protective fungicide applications from flower bud emergence to harvest [29].

### 3.4. Sclerotinia sclerotiorum

*Sclerotinia sclerotiorum* is a necrotrophic true fungus capable of infecting a wide array of crops globally [82]. It is the causative agent of multiple symptoms on aerial plant organs, including white mold, watery soft rot, cottony rot, drop, crown rot, and *Sclerotinia* rot. It is a true generalist, lacking any apparent host specificity, and is capable of infecting crops in temperate, tropical, and arid regions [83,84,85,86,87]. Losses caused by this phytopathogen vary significantly based on geographic location. Temperate climates are particularly susceptible, where yield losses reliably surpass 20–35% [82].

**Figure 3:**
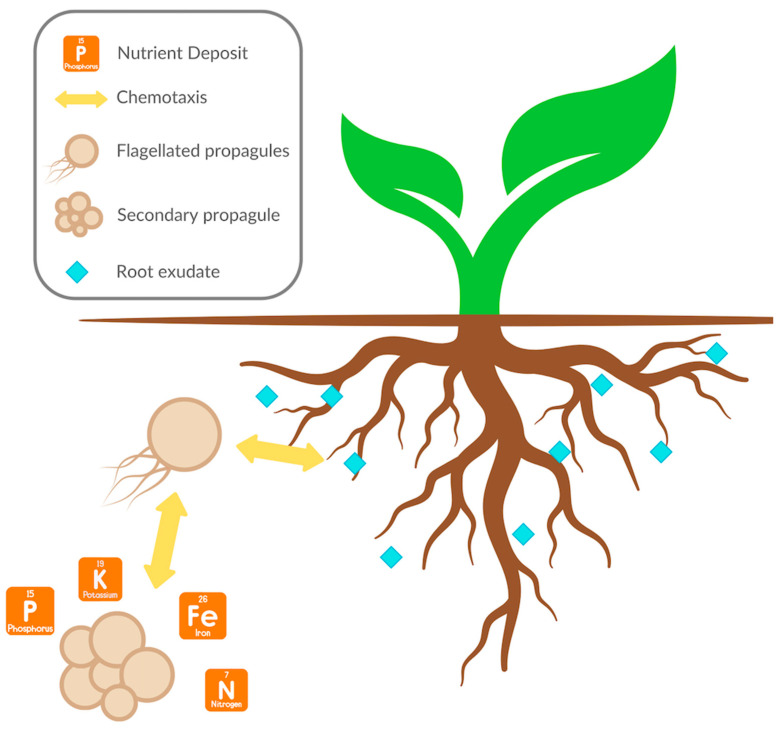
Germination of phytopathogenic propagules that target root systems. Spore germination does not always occur on plant roots. In most cases, the spores are drawn through chemotaxis to nutrients, which can be mineral deposits in the soil, growth media or exudates from plant roots. In the case of root exudates, the abundance of nutrients allows for germination and mycelium development, hence further colonization of the host. In the case of mineral deposits, the lack of a host can lead to a quick exhaustion of nutrients, such as phosphorous (P), potassium (K), iron (Fe), and nitrogen (N), and the development of ‘’secondary’’ propagules to persist in the environment until favorable conditions are met.

In hydroponics, *S. sclerotiorum* is commonly observed in solanaceous and cucurbit crops [30], where it can either infect the roots and stem or the floral organs of the host [88]. The site of infection depends on the type of propagule that is initiated. Mycelium forms during direct germination of the sclerotia, possibly induced by chemotaxis with the root exudates of the plant (Figure 3). The fungus can infect the roots and the stem, causing severe wilt or death [89]. Affected leaves start wilting and become grayish before turning brown [30]. The stem of the infected plants harbors white mycelia and sclerotia, which differentiates it from other root or stem rot diseases [30]. In the case of indirect germination, the sclerotia form multiple stipitate apothecia, which generate ascospores that are forcibly expelled into the air to reach more favorable organs for germination [89]. This is the main infection vector of aerial plant organs [90], whereby spores landing in the sugary secretions of the floral organs germinate and colonize adjacent tissue, causing leaf and stem rot. Furthermore, carried contaminated soil can be the transmission vector into a hydroponic greenhouse through equipment or staff (carts, shoe soles, clothing). Additionally, sclerotia can survive in plant debris for up to five years [91] and can be transmitted through moisture in the greenhouse. 

Despite the dual mode of infection of the host plant, *S. sclerotiorum* is not capable of initially colonizing living tissue. Whether in the mycelial or ascosporic form, this fungus can only infect and colonize dead organic matter or senescent tissue, where it feeds saprophytically to support its invasion of healthy live tissue [92,93]. If *S. sclerotiorum* is not able to curb the immune system of the plant during the biotrophic phase, an HR by the plant can collapse tissue around the fungus and trap it in a benign form, preventing the fungus from infecting the rest of the plant [94]. A successful transition to the fungal necrotrophic phase exhibits the production of more complex feeding structures called infection cushions. It is from these structures that secondary necrotrophic hyphae are produced to penetrate living tissue and liberate cell death-inducing virulence factors such as CWDEs and OA. It must be noted that environmental conditions favoring this switch are temperature, moisture, and pH [95,96,97]. Interestingly, the secretion of OA decreases the apoplastic pH to approximately 4.0, promoting the formation of new infection cushions, acting in a positive feedback loop [98]. Upon initiation of host tissue necrosis, new sclerotia are formed to support the next generation of pathogens [99]. It is due to this cumulative process that *S. sclerotiorum* is considered an aggressive necrotroph. 

Contaminated soil is one of the main transmission vectors for *S. sclerotiorum*; hence, hygiene of equipment and staff clothing needs to be maintained [100]. Frequent sterilization of growth media, buckets, shovels, etc., is also recommended given the capacity of sclerotia to survive on them. Additionally, as a fungus, it thrives and propagates in environments with high moisture and humidity. Therefore, controlling the microenvironment around the plants is conducive to disease management. Appropriate spacing between plants to increase air circulation, decrease nutrient solution dripping onto adjacent plants, and reduce plant-to-plant contact can limit disease propagation [82]. Other humidity control methods, such as cool air exchange and mulching, can reduce disease incidence by limiting the moisture in the greenhouse. Finally, *S. sclerotiorum* can also survive on infected plant debris for long periods of time, where it continues to produce spores. Hence, effective removal and disposal of plant debris and residue is important [100].

### 3.5. Phytophthora

*Phytophthora* spp. are oomycetes and not true fungi, based on distinct physiological differences (Figure 1), including primarily diploid life stages, cellulose-based cell walls, and biflagellate oospores [101,102]. Currently, the taxon contains more than 200 accepted species [103,104] with the number of known species expected to rise in the future. The genus contains several of the most historically impactful phytopathogens, including the causative agent of the potato blight, *Phytophthora infestans,* that was responsible for the 19th-century Irish potato famine. *P. infestans* and *P. capsici* are the most common pathogens in hydroponic systems, inducing disease in Cucurbitaceae and Solanaceae, causing a range of symptoms including damping off, stem and vine blight, wilting, and fruit rot [105]. 

*Phytophthora*’s lifecycle typically begins as oospores [106]. Oospores are robust, can survive for many years, and are resistant to cold temperatures and desiccation [107]. Oospore germination causes sporangia to form, which are spread by wind and water to directly infect host tissue. In wet conditions, however, oospores can germinate into motile zoospores that “swim” towards host tissue to infect [108]. In protected greenhouses, spore germination and disease development are optimal at high humidity (upwards of 75%) and at temperatures of 28–30 °C (*P. capsici*) or 20 °C (*P. infestans*), depending on the species [29]. *Phytophthora* disease progression is usually associated with higher temperature and moisture [109]. In a recent report in Korea [110], *Phytophthora* DNA was detected in hydroponic nutrient wastewater from facilities growing tomatoes, paprika, and strawberries. These are the most hydroponically cultivated crops in Korea, and strawberry growing facilities exhibited the highest rate of *Phytophthora,* suggesting the need for nutrient solution sterilization when recycling to reduce the risk of disease persistence in the facility.

Both *P. infestans and P. capsici* are necrotrophic, causing host cell death; this is preceded by a biotrophic phase in which the fungi feed on living tissues (Figure 2) [111]. *Phytophthora* spp. have the capacity to modulate host immune responses. For example, *P. infestans* can suppress programmed cell death in host tomato plants through the secreted suppressor of necrosis 1 protein (SNE1) [112]. Production and the subsequent secretion of these effector proteins take place in contact structures called haustoria [113]. *Phytophthora* pathogens have an arsenal of virulence factors, such as the cell wall-degrading pectin methylesterase [114], to induce host cell necrosis, causing the lesions that constitute blight symptoms. In a recent study, researchers reported the characterization of a new small cysteine rich protein (SCR82) from *P. capsici* involved in the regulation of the oomycete pathogenicity [115]. This SCR seems to be specific to *Phytophthora* oomycetes, and it is upregulated during infection in comparison to its production levels in mycelial, sporangia, and zoospore stages. Interestingly, disease severity in infection models were affected by SCR82, with the knockout mutant causing significantly smaller lesions on *Nicotiana benthamiana* (a close relative of tobacco) leaves and bell pepper stems. Interestingly, ectopic expression of *scr82* and its injection into leaves caused programmed cell death (PCD) but only in tomato leaves. These data suggest that SCR82 may be host specific. SCR82 was also able to induce multiple plant immune responses in tomato leaves by triggering ROS burst, callose deposition, and the induction of 19 defense-associated genes in tomato leaves in a PAMP-dependent manner. This type of interaction was also observed in another *P. capsici* effector, PcAvh1, in bell pepper, tobacco, and tomato hydroponic infection models [116]. Much like the previous study, this effector was upregulated during the infection, in comparison to other life stages. Knockout mutants exhibited significantly reduced disease severities in all three plant species. However, unlike the previous study on SCR82 [115], ectopic expression of PcAvh1 induced PCD in all the tested plants. Due to its wide range of virulence and survival factors, management of *Phytophthora* is currently limited. At the present time, there is no commercially available *P. capsici*-resistant chili cultivar [117,118]. It is best to avoid capsicum cultivation in severely affected greenhouses. Fungicide resistance has been reported in multiple strains, decreasing the efficacy of fungicide treatments in disease control [117,119]. However, some measures can be taken to either reduce the incidence of the disease or its severity. For instance, using certified healthy seeds from disease-free areas can decrease the probability of disease emergence [29]. Similarly, managing both temperature and moisture can decrease the severity of the disease; 30 °C and high humidity are ideal for *P. capsici* oomycetes [29,120]. 

## 4. Phytopathogens Targeting Roots and the Vascular System

### 4.1. Fusarium oxysporum

The genus *Fusarium* contains many true ascomycete fungi, including the phytopathogen *Fusarium oxysporum* [121]. Like many true fungi, *F. oxysporum* strains produce a variety of pigments, have chitin-based cell walls, and have a predominantly haploid life cycle (Figure 2) [122,123,124]. Multiple host-specific *F. oxysporum* strains exist that were assigned to formae speciales (f.sp) following the proposal by [125], which is still used today. For instance, *F. oxysporum* sp. *lycopersici* only infects tomato plants, *F. oxysporum* sp. *cucumerinumon* only infects cucumber plants, and *F. oxysporum* sp. *fragariaeon* only infects strawberries. 

*Fusarium* is an opportunistic pathogen due to its ability to exist without infecting surrounding plants [126,127]. This fungus has high host specificity and colonizes dead organic matter and plant roots, suggesting both substrates are sufficient for its reproduction, yet it is unclear whether *F. oxysporum* is capable of sexual reproduction. In hydroponics, *F. oxysporum* causes fusarium wilt in pepper and tomato plants. It can survive in coco coir (coconut husk fiber) and rockwool blocks while thriving in temperatures between 25 and 30 °C. With the exception of *F. oxysporum* f. sp. *radicis-lycopersici*, which thrives at lower temperatures, i.e., 10–20 °C and causes crown rot in tomatoes [29]. Disease progression can occur in a wide range of humidity conditions, but high humidity is important for *Fusarium* spore germination [29]. In a recent report [110] in Korea, fungal incidence was measured in hydroponic nutrient wastewater from tomato, paprika, and strawberry-growing greenhouses. Most facilities (64%) presented *Fusarium* DNA in their nutrient solution waste, with strawberry greenhouses exhibiting the highest quantities of fungal DNA. Given the specificity of *Fusarium* species, it remains unclear if all DNA detected is that of pathogenic species or naturally occurring *Fusarium* endophytes. At the very least, this study shows that Fusarium species can survive in nutrient solution across a growing cycle in hydroponics operations for tomatoes, paprika, and strawberry plants.

Typical fusarium wilt symptoms include yellow and stunted plants, progressing to death in younger plants and seedlings [30]. The fungus grows inside the plant xylem, where it clogs it and siphons off nutrients directly from the vascular system [128]. The infection cycle commences from spores that germinate in the presence of root exudates, as depicted in Figure 3. In hydroponic systems, *Fusarium* germination was observed to be highly crop specific. Whereby *F. oxysporum* special formae do not exhibit the same rate of germination and hyphae growth when exposed to root exudates from different plants. For example, *F. oxysporum* f. sp. *lycopersici* exhibited a significantly higher germination rate in the presence of root exudates of its specific crop (tomato) in comparison to root exudates from bean, barley, tobacco, and cucumber [129]. In this study, the authors measured the germination rate of microconidia with sterile filtered root exudates from plants of varying age. They observed that both tomato and sweet pepper root exudates exhibited the highest germination rates of 44.9% and 35.8%, respectively. The age of the plant supplying the root exudates also influenced the rate of germination, with the lowest rates recorded for the youngest plants. Microconidia germination was highest in root exudates from 40- and 70–90-day-old tomato plants. Surprisingly, root exudates from 50–60-day-old plants decreased germination rates to 2–3.5%, suggesting growth of the plant and accumulation of exudates is not the sole factor necessary for germination but potentially the composition of the exudates. Further analysis revealed that for the tested *F. oxysporum* f. sp. *lycopersici* strains Fol and Forl, their microconidia germination rates increased significantly when the root exudates were combined with water-insoluble polyvinylpolypyrrolidone (PVPP), regardless of the plant root exudate source. PVPP is a known flavonoid and phenolic compound sequester [130], which suggests that these root exudate compounds [131] have an inhibitory effect on *F. oxysporum* f. sp. *lycopersici* germination. It must be noted that in nature, *F. oxysporum’s* main form, in the absence of a host, is believed to be its chlamydospores due to their superior persistence capacity in comparison to its mycelial or microconidia form [127]. Another study determined that *F. solani* microconidia exhibited increased germination in minimal media supplemented with flavonoids [131], further highlighting the specific nature of host–pathogen interactions in *Fusarium*. Not only are *Fusarium* species sensitive to the root exudates of specific crops, but they can also alter the root exudates to affect the germination rates of other *Fusarium* species [132]. Indeed, researchers observed that root exudates from tomato plants infected with a non-pathogenic *F. oxysporum* reduced the germination rates of microconidia of the pathogenic strains *Fol* 007 and *Forl* 101587.

Successful germination of *Fusarium* spores does not always translate to a symptomatic infection of the host plant because of the protection provided by the plant’s immune system. Plant immune systems can deny access to the xylem by thickening their epidermal walls or HR collapsing root cells. This effective immune response may explain seemingly biotrophic interactions observed between non-pathogenic special formae of *F. oxysporum* and the host plant [127]. Indeed, even necrosis of subepidermal cells was observed for non-pathogenic *F. oxysporum* strains [133]. Spores migrate towards sources of nutrients and germinate upon root contact to form primary hyphae. The fungus grows on the root surface before gaining entry through penetrative appressoria [127]. Entry into the plant roots and the subsequent growth towards the vascular system of the plant can occur intracellularly in the apoplast or intracellularly via the production of cell wall-degrading enzymes [134,135]. Reproduction and formation of new spores take place in the xylem of the infected plant, where the spores can be transported to new healthy, uninfected tissue after host death [136]. At this point the plant vessels start to get clogged, and the fungus starts to produce toxins such as fusaric acid, dehydrofusaric acid, and lycomarasmin [136]. Both the lack of nutrients from the vessel clogging and the toxins contribute to the wilt symptoms of the infected plant [16,137]. Toxins can migrate to the leaves, where they reduce chlorophyll synthesis along the veins (vein clearing), thus resulting in reduced photosynthesis, leaf cell membrane permeability disruption, and loss of moisture trapping. Subsequently, leaf epinasty, wilting, interveinal necrosis, browning, and ultimately death of the whole plant can occur [16]. *F. oxysporum* sp. *lycopersici* produces high levels of the non-specific toxin fusaric acid (FA) which can contaminate and then accumulate in infected cereals and corn; it is toxic to human and animal consumers [138]. In one study, FA infiltration in tomato leaves reduced both the amount of chlorophyll and the subsequent photosynthesis by up to 5-fold, which was accompanied with leaf browning and necrosis. [136] Additionally, the presence of H_2_O_2_ in FA-treated leaves was increased to 4.5 μM/g compared to 0.5 μM/g in control plants, confirming the induction of oxidative stress by FA.

Once the host plant dies, *Fusarium* can survive in a saprophytic state, feeding on dead tissue and later sporulating and producing asexual spores [127]. The resulting chlamydospores are highly resistant to abiotic stresses and can survive indefinitely in the absence of a host [139,140], making *F. oxysporum* contamination difficult to manage. Most fusarium wilt symptoms are first observed as a slight vein clearing on outer leaflets and drooping of leaf petioles. As the disease progresses, the older leaves wilt, turn yellow, and become necrotic. These symptoms, in many cases, initially appear on one side of the stem and progress upward until all foliage wilts, the stem dies, and infected fruits can rot and drop off from the plant [141]. In a cross-section of the stem near the base of the infected plant, a brown ring is evident in the area of the vascular bundles. The upward extent of the discoloration depends on the disease severity. Since *Fusarium* is a ubiquitous pathogen, present in highly persistent chlamydospore forms, the most effective disease prevention method is to use resistant cultivars [29]. Indeed, these chlamydospores can persist in growth substrates, dead tissue, contaminated water, and contaminated equipment. Hence, extensive hygiene practices and periodic pruning are important for limiting disease incidence [141].

### 4.2. Pythium

The genus *Pythium* contains species that are pathogenic to plants [142], animals [143], algae [144], and other fungi [145]. *Pythium* spp. are oomycetes closely related to *Phytophthora*. More than 370 species of *Pythium* spp. have been identified to date (mycobank.org). Symptoms of a *Pythium* infection include damping-off, root rot, collar rot, soft rot, and stem rot in multiple agricultural systems, including nurseries, greenhouses, and agricultural fields [146,147,148]. *Pythium* spp. are naturally found as either a propagule (zoospores, oospores, or sporangia) or in the mycelial form. The oospore is the primary survival and infection structure and can stay viable for long durations [149]. The survivability of *Pythium* propagules relies on finding suitable conditions for reproduction [150]. In the absence of optimal conditions, *Pythium* spp. form secondary propagules [151]. Germination of primary or secondary propagules is triggered by exogenous stimuli, usually root exudates (Figure 3), which leads to the formation of hyphae and subsequent penetration and colonization of host tissue [152]. Sporangia can also infect living tissue directly through hyphal tube germination or by producing zoospores in the presence of high moisture conditions [152]. Furthermore, *Pythium* spp. can also be found as mycelia in the soil, where they survive saprophytically and colonize fresh organic substrate [152]. 

In hydroponic systems, *Pythium* spp. can be introduced in multiple ways, including plug transplants, soil, growing media, plant refuse, and irrigation water. Additionally, *Pythium* can be introduced via greenhouse insects, including fungus gnats and shore flies [153]. These small insects feed on fungi and algae, respectively, inadvertently spreading fungal diseases throughout a greenhouse. Although adult fungal gnats can carry double the number of fungal pathogen spores externally, larvae ingest 10–20 times more spores, which are viable and capable of infecting crops [154]. Entomological vectors can potentially be mitigated by using a fungal parasite to exert control, such as the fungus Clonostachys rosea, to reduce spore viability in insects. Once inside the greenhouse, *Pythium* causes different symptoms based on the growth phase of the affected plants. Pre-emergence damping-off refers to seeds rotting before they emerge, while post-emergence damping-off refers to the death of newly emerged seedlings [153]. Indeed, the release of exudates and imbibing of water by seeds during their germination phase makes them an ideal target for *Pythium* infection [152]. In young plants, *Pythium* initiates crown and root rot, causing the plants to wilt when they start producing fruit [153]. Furthermore, according to [110], *Pythium* is able to persist in growing nutrient solution in hydroponically grown tomatoes, paprika, and strawberry plants. It remains unclear whether the plants harvested exhibited severe disease phenotypes. However, the goal of the study was to determine the incidence of fungal and oomycetes in waste nutrient solutions of hydroponic greenhouses.

In the case of an infection, initial symptoms appear as brown to dark-brown lesions in the root systems. As the disease progresses, brown, soft, stubby roots that lack feeder roots begin appearing, and the root cortex starts to peel away, leaving the string-like vascular bundles underneath [153]. Furthermore, *Pythium* can also affect the stem of the plants, where it causes water-soaked lesions with soft rot at the base. In greenhouse-diseased cucumbers, these lesions take on an orange-brown color [153].

Conditions favorable to the development and spread of *Pythium* disease tend to vary for each species. The moisture, temperature, organic matter content, pH, and soil type all influence germination and disease progression [155,156]. High moisture supports the motility of zoospores and increases the size of the spermosphere, which play a crucial role in infection by *Pythium* propagules [152]. Indeed, high moisture content is correlated with higher disease incidence as well as root rot severity [157,158]. Additionally, higher temperatures (25–30 °C) have been linked to increased severity in chili plants [159]. *Pythium* spp. Contamination, including *P. graminicola*, *P. myriotylum*, and *P. aphanidermatum,* is prominent in hydroponic growing operations that do not use optimized temperature control [160,161]. 

Multiple studies have been conducted to elucidate factors influencing *Pythium* disease progression. Researchers compared the optimal conditions for disease development in soybean cultivars infected with different *Pythium* spp. [162]. Disease control was tested at 4, 12, 20, and 28 °C. Temperature did not seem to affect the disease progression in *P. ultimum*, which exhibited the highest disease score at all temperatures. In contrast, *P. aphanidermatum* exhibited an increase in disease score with an increase in temperature; seed rot scores were observed to be 65.4% at 20 °C and 85.4% at 28 °C. These findings were also in agreement with an earlier study [163], where it was noted that *P. aphanidermatum* root rot severity correlated positively with temperature (up to 30 °C) and pH (up to 7.0) in both vermiculite and peat-grown poinsettia. Despite these studies highlighting the importance of the interactions between *Pythium* spp., host plants, and environmental conditions, the genus lacks many of the specialized virulence factors present in other phytopathogens [164]. For instance, *Pythium* lacks RxLR effectors with avirulent activities unlike other phytopathogens [165,166]. These are Arg-any amino acid-Arg-Leu cytoplasmic effectors expressed by phytopathogens and have a wide range of host process manipulation. About 50% of all identified RxLR effectors are negative host immunity regulators. Subsequently, there are no *Pythium*-resistant cultivars commercially available to growers, but there are some variants that exhibit disease tolerance [153]. These findings align with the lack of specific effectors in *Pythium*’s virulence arsenal, since tolerance is usually defined as non-specific interactions leading to an increase in plant survival, which can stem either from a population or individual effect [167]. Despite sharing many genomic similarities with *Phytophthora* spp. when it comes to pathogenicity factors, *Pythium* species exhibit a lack of cutinases, pectinases, and xylanases, which are CWDEs facilitating cell host penetration and necrosis [168]. Even when present, *Pythium*’s expression level of cellulases and pectinases is low compared to other oomycetes. The presence of plant starch and sucrose-degrading enzymes such as α-glucosidase, α-amylases, α-glucoamylases, and invertases can explain the propensity of *Pythium* spp. to target younger plants with no secondary growth, because the phytopathogen lacks the genetic arsenal to effectively penetrate older tissue to metabolize its nutrients [169].

To manage *Pythium* risks in hydroponic systems, it is important to control the vectors of its transmission and the environmental conditions favorable to disease development. *Pythium* is particularly adept at infecting young plants; thus, germination and transplanting steps are key in managing disease risk. It is important to obtain uninfected seeds and maintain optimal sanitation during the transplantation phase [153]. Careful manipulation of young seedlings and transplants is recommended to minimize tissue damage, which can increase the probability of disease introduction. Additionally, recurrent sterilization of growth media and growth substrates is mandatory. Furthermore, to curb disease growth conditions, it is best to avoid low light levels, low pH, high salt concentrations, and warm growing conditions. For example, for cucumbers, the optimal pH level of nutrient solution for disease control is 5.0, before the pH is adjusted, after five weeks of growth, to 5.8–6.2 [170]. 

## 5. Beneficial Fungi

**Figure 4:**
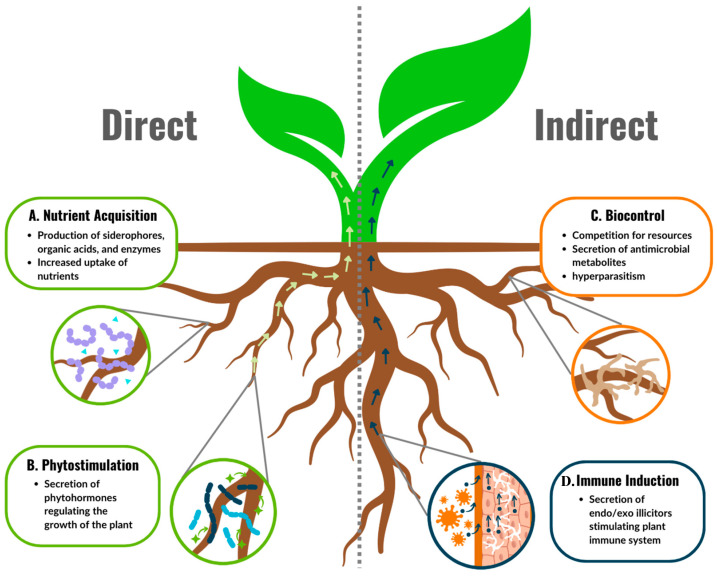
Plant growth-promoting mechanisms used by fungi (i.e., PGPF) fall under two broad categories: direct and indirect methods. Methods that directly benefit the plant are considered direct methods (A, B). In contrast, methods that benefit the plant indirectly, by decreasing the deleterious effects of pathogens are considered indirect methods (C, D). PGPF may secrete organic acids, siderophores, or enzymes to increase the bioavailability of nutrients such as iron, phosphorous, and potassium (A). Phytohormones (e.g., indole-3-acetic acid (IAA), cytokinin, and gibberellins) produced by PGPF can be taken up via the roots and stimulate growth and regulate stress within the host plant (B). PGPF may also participate in antagonistic relationships with phytopathogens in the rhizosphere, thereby controlling phytopathogen populations and decreasing infection and disease severity for the host plant (C). Many PGPF also secrete secondary metabolites that function in signaling cascades within the plant, inducing plant defenses and preparing the plant to encounter phytopathogens (D). Both phytohormones and immune-inducing metabolites taken up by the roots can function throughout the plant.

Plants have coevolved with fungi for millions of years, leading to a range of plant-fungal relationships varying from symbiosis to pathogenicity [171]. Fungi such as *Aspergillus*, *Penicillium*, *Talaromyces*, and *Trichoderma* have co-evolved a mutually beneficial relationship with plants. These organisms have developed a multitude of intricate tools to benefit plants (Figure 4), akin to the diversity of strategies that bacteria use to facilitate plant growth [172]. PGPF actively promote the health of the host plant through diverse mechanisms, including (i) biocontrol, (ii) immune system induction, (iii) phytostimulation, and (iv) nutrient uptake. The role of bacteria in hydroponic growth promotion and biocontrol has been extensively studied and reviewed [6,173]; the role of fungi in hydroponic systems is understudied in comparison. Therefore, this review presents recent hydroponic-based research as often as possible and supplements with soil-based research where necessary; all referenced studies were performed in CEA systems. The following hydroponic studies demonstrate that *Aspergillus* [174,175], *Penicillium* [175,176,177,178,179], *Talaromyces* [180,181], and *Trichoderma* [182,183,184] species can persist in hydroponic systems and provide support for the beneficial role of PGPF in hydroponic crop production.

Biocontrol is the capacity of an organism to control and suppress the growth of pathogens on the host. This can be affected by a number of different mechanisms, including producing antifungal peptides or by occupying the same niche as the pathogen, hence outcompeting the pathogen for the same resources [185]. Immune induction refers to the capacity of fungi to promote the activation of a plant immune response, allowing the plant to successfully resist a phytopathogen infection before visible symptoms appear [186]. Supplemental Table S1 summarizes the large body of literature on PGPF with biocontrol ability and immune induction capacity. While this biocontrol section predominantly focuses on the ability of fungi to promote growth via control of fungal phytopathogens, it should be noted that many PGPF are also capable of controlling plant pathogens from other domains of life through these mechanisms [185,187,188].

Phytostimulation is the ability of the fungus to stimulate the production of phytohormones that assist the plant in regulating either its growth cycle and/or abiotic and biotic stresses. Biotrophic pathogens sometimes employ this strategy to promote the growth of the tissue they are feeding on. Both PGPF and biotrophic pathogens can produce and modulate the levels of phytohormones in their host, such as IAA [189]. Lastly, nutrient uptake is the ability of fungi to metabolize environmental nutrients into easily accessible forms by the host plant. These include the macronutrients nitrogen, phosphorus, and potassium, as well as the micronutrients iron and zinc. These nutrients are often limiting in soil, or exist predominantly in forms that are not bioavailable for uptake by plants. Nitrogen is metabolized from atmospheric N_2_ into ammonia by soil microbes. Iron is generally chelated from the environment via microbial siderophore production; indeed, these secondary metabolites can allow for the culture of “unculturable” bacteria. This phenomenon has been one of the explanations for the plate count anomaly [6]. Phosphate is generally made more bioavailable for the host plant by microbial phosphate-solubilizing organic acids and enzymes [190]. Supplemental Table S2 summarizes examples of direct plant growth promotion by PGPF. 

## 6. Plant Growth-Promotion Through Biocontrol and Immune Induction

### 6.1. Aspergillus: Biocontrol and Immune Induction

The genus *Aspergillus*, within the Ascomycota division, encompasses six subgenera and over 339 accepted species. Many *Aspergillus* species have applications in biotechnology, cellular biology, human health, and agriculture [191]. For example, several *Aspergillus* species are commercially important in the production of fermented foods, citric acid, and antimicrobial compounds [192]. Beyond commercial applications, *Aspergillus* species are naturally ubiquitous in both nutrient-rich and nutrient-deficient aerobic environments, including the hydroponic rhizosphere [193]. *A. niger*, *A. flavus*, *A. fumigatus*, and several other *Aspergillus* species are known endophytic PGPF that promote growth of hydroponic crops and act as biocontrol agents against phytopathogens [194,195]. The most common mechanisms of biocontrol include competition for an identical niche and induction of systemic resistance. In addition, some of these strains have been reported to produce siderophores, antifungal metabolites, ammonia, hydrogen cyanide, and hydrolytic enzymes [194,196,197]. 

Several in vitro studies report the ability of *Aspergillus* species to control fusarium wilt in tomatoes caused by *F. oxysporum* infection [194,195]. In agar well diffusion assays, the crude extracts from *A. flavus*, *A. fumigatus*, and *A. nidulans* all produced significant zones of inhibition in a lawn of *F. oxysporum* [194,195]. Using a similar assay, the minimum inhibitory concentrations (MICs) of *A. niger* and *A. flavus* extract against *F. oxysporum* were 0.25 mg/mL and 0.50 mg/mL, respectively [198]. The results of these assays indicate the production of antagonistic metabolites by *Aspergillus* that are effective against *F. oxysporum*. 

In addition to in vitro testing, *Aspergillus* strains were found to alleviate the severity of fusarium wilt in plant trials when the PGPF candidates were inoculated before and simultaneously with *F. oxysporum*. Researchers observed an 86% decrease in disease severity when *A. fumigatus* was inoculated prior to infection with *F. oxysporum*, relative to infected controls [195]. Disease severity decreased from 88% for the infected control to 17% and 38% for *A. niger*- and *A. flavus*-inoculated treatments, respectively [198]. Inoculation with *A. flavus*, *A. nidulans*, and *A. fumigatus* as a consortium decreased disease severity from 84% for the infected control to 38%, 25%, 34%, and 13%, respectively [194]. Interestingly, *A. fumigatus* and *A. flavus* inoculation in the absence of *F. oxysporum* increased shoot and root growth relative to the healthy control [194,195]. This indicates that *A. fumigatus* and *A. flavus* employ PGPF mechanisms beyond biocontrol, which will be discussed in detail later in this review.

The induction of pathogen resistance in plants by *Aspergillus* was also observed to occur via salicylic acid (SA), jasmonic acid (JA), and ethylene (ET) signaling pathways of induced systemic resistance (ISR) and systemic acquired resistance (SAR). For example, an upregulation of the *PR1* gene occurs in tomato plants inoculated with *A. terreus* and then challenged with *P. syringae* pv. *tomato* DC3000, the causative agent of bacterial speck disease [186]. Inoculated tomato plants experienced significantly decreased bacterial speck infection compared to the infected control [186]. The *PR1* gene codes for pathogenesis-related protein 1, and its expression is a marker of SA-mediated induction of SAR [199]. Furthermore, *A. tubingensis*, *A. alabamensis*, and *A. oryzae* demonstrated significant antifungal activity against *F. oxysporum* in both in vitro and pepper plant trials. The mechanisms used by these species include increasing antioxidant enzyme activity in the plants, producing HCN to negatively impact pathogen proliferation, and interfering with the cell wall integrity of *F. oxysporum*. All three strains also tested positive for siderophore production [200]. *Aspergillus* inoculation has also been linked to decreased oxidative stress via increased antioxidant activity in tomato plants. Plants inoculated with *A. flavus*, *A. nidulans*, and *A. fumigatus* either singly or in combination had significantly elevated peroxidase (POD) and polyphenol oxidase (PPO) levels, which are known plant antioxidants produced to alleviate biotic and abiotic stresses [194,201,202]. 

Overall, the ability of fungi to biocontrol nematodes is not well studied, and the mechanisms are not yet well understood. One recent study provides a compelling case for further research in this sector [203]. The inoculation of eggplant seedlings with *A. oryzae* decreased the observed number of female *Meloidogyne incognita*, a root-knot nematode phytopathogen, by 95% and the number of egg masses by 80%. The study attributes this observation to hydrogen cyanide (HCN) and toxic volatile organic compound (VOC) production by *A. oryzae*, inhibiting *M. incognita* proliferation [203]. Until this topic is more thoroughly investigated, other pest management strategies, such as chemical nematicides and crop rotation, will continue to be the nematode control strategy of choice for most growers [204].

### 6.2. Penicillium: Biocontrol and Immune Induction

*Penicillium* is a genus of fungi within the Ascomycota division that includes 354 accepted species. These species inhabit a wide range of environments, including those with both moderate and extreme temperatures, high salinity, and high acidity [205]. The most well-characterized member of the genus is *P. chrysogenum*, the source of the antibiotic penicillin [206]. Other species of *Penicillium* have economic value for their ability to produce antimicrobials, remediate contaminated soils, and promote hydroponic plant growth as root endophytes [176,205]. 

*Penicillium* spp. act as biocontrol agents against bacteria, fungi, nematodes, and viruses [177,207,208,209,210]. In fact, biocontrol studies constitute a large proportion of recent studies on PGPF *Penicillium* species due to the vast amount of antibacterial and antifungal secondary metabolites produced and secreted by *Penicillium* species [185]. Overall, the general mechanisms of *Penicillium* biocontrol include competition for the same niche, the induction of systemic resistance, and antibiosis. 

Multiple *Penicillium* spp. are reported biocontrol agents (BCAs) of the bacterial plant pathogen *P. syringae* pv. *tomato* DC3000, the causative agent of bacterial speck disease. Thus, scientists observed decreases in disease severity when *P. viridicatum* was used as a biocontrol agent of *P. syringae* pv. *tomato* DC3000 of hydroponically grown *A. thaliana* [211]. The reported mechanism was the induction of ISR via ethylene signaling. Another study observed the ability of *P. simplicissimum* to also interfere with *P. syringae* pv. *tomato* DC3000 infection of *A. thaliana* [177]. The results of fungal inoculation were like the results of exogenous abscisic acid (ABA) application; in treated plants, the expression of *MYB44*, an enhancer of the ABA signaling pathway, was altered, and stomatal opening was restricted. The stomata are the entry point by which *P. syringae* infects plants [212]. Thus, PGPF-mediated restriction of the stomatal opening, because of ABA-mediated induction of systemic resistance, may be a successful strategy of biocontrol against *P. syringae* and subsequent bacterial speck disease in hydroponic systems [177]. 

In addition to its capability as a BCA of bacterial phytopathogens, *P. simplicissimum* has also been reported to be a BCA of viral infection of plants. Researchers observed that inoculation with *P. simplicissimum* decreased accumulation of cucumber mosaic virus particles in *A. thaliana* and decreased disease severity compared to infected controls [213]. The study credits these observations to the robust induction of systemic resistance via SA-, JA-, and ET-mediated signaling pathways. Reverse transcriptase PCR results confirm the upregulation of SA-responsive genes *PR-1*, *PR-2*, and *PR-5*, ET-responsive gene *HEL*, and ET/JA-responsive gene *PDF1.2* in inoculated plants, compared to the infected control [213]. Similarly, *P. simplicissimum* is reported to be an effective BCA against papaya ringspot virus infection (PRSV) in cucumbers by upregulating defense-related genes, including *PR-1* and PAL genes [207]. While both studies provide evidence for the effective biocontrol of viruses by *Penicillium* species, further research is required to elucidate the exact fungal component that triggers the immune induction. 

Like *Aspergillus*, *Penicillium* species have also been linked to the biocontrol of nematodes, but further research is required to clarify the intricacies of the fungi–nematode interaction and solidify the literature understanding. *P. chrysogenum* is an effective biocontrol agent of the root-knot nematode *M. incognita*. Application of the fungus decreased nematode invasion rate by 67% and nematode reproduction by 69% in greenhouse cucumbers; it also significantly decreased gall formation and mass of nematodes per gram of cucumber root [209]. A subsequent study by [208] revealed that *P. chrysogenum* inoculation increased the expression of both SA- and JA-mediated defense genes, including *PR-1*, *PAL-1*, and *CHIT-1*, as well as several transcription-factor-related *MYB* genes and cell-wall modification genes *EXT2*, *FH20*, and *PE*. The differential expression of these genes can be linked to the induction of systemic resistance and thus the observed decrease in nematode infection [208]. 

Importantly, *Penicillium* species are effective BCAs of various fungal pathogens of hydroponic crops, including *C. orbiculare*, *F. oxysporum*, *Rhizoctonia solani*, and *S. scleotiorum* [198,214,215]. *P. oxicalum* and *P. rubens* have both demonstrated biocontrol ability against *F. oxysporum* in tomatoes, and *P. expansum* has conferred similar biocontrol of the fungal pathogen in peppers. *P. oxicalum* decreased disease severity via the induction of ISR [198]. [176] revealed upregulation of genes of the xylanolytic system in *P. rubens*, including the *XlnR* transcriptional regulator. This supports the current theory that the xylanolytic system of *P. rubens* is involved in the induction of systemic resistance in plants, conferring protection against *F. oxysporum*, although the intricacies of the phenomenon remain unclear [176]. *P. expansum* decreased fusarium wilt disease index in peppers, which was attributed to its capacity of producing HCN and other antifungal compounds [216]. *P. commune* exerted biocontrol over the fungal pathogen *S. scleotiorum*, the causative agent of white mold, in eggplants. The biocontrol agent decreased disease severity from 94% in the infected control to 9% in treated plants. Notably, *P. commune* performed better than the commercial fungicide, M-Topsin, which only decreased disease severity to 50%. *P. commune* produces antimicrobial secondary metabolites, and in vitro antagonism assays revealed 86% inhibition of *S. scleotiorum* growth [214]. 

In many of the biocontrol studies mentioned thus far, antagonism against the pathogen is demonstrated in vitro using disk or agar-well-based plate assays. In several cases, the fungal extract alone is sufficient to inhibit pathogen growth; this is evidence that the biocontrol mechanism is antibiosis and involves the production of antimicrobial metabolites or extracellular hydrolytic enzymes that retain antagonistic activity in the absence of the source fungus [194,198,210]. In contrast, another study did not observe inhibition of *R. solani* or *C. orbiculare*, the causative agents of damping-off and anthracnose, respectively, by the PGPF *P. viridicatum* in in vitro assays [215]. Nevertheless, they observed 35–74% and 43–58% decreases in the severity of damping-off and anthracnose, respectively. This study posits that the biocontrol mechanism in this case is more likely the induction of ISR, occupational biocontrol, and competition [215]. Inoculation with *P. viridicatum* increased shoot fresh weight by 46% and root fresh weight by 26% compared to the infected control, indicating that non-antibiosis biocontrol mechanisms can be effective strategies by PGPF. 

### 6.3. Talaromyces: Biocontrol and Immune Induction

*Talaromyces* is a genus of ascomycete comprising over 170 accepted species within the family *Trichocomaceae.* These fungi are predominantly rhizospheric, with many species forming endophytic associations [217]. Many species within this genus, including *T. wortmannii*, *T. flavus*, *T. pinophilus*, and *T. apiculatus,* have been identified as effective PGPF based on their abilities in biocontrol, nutrient solubilization, plant hormone synthesis, and induction of host defenses [218,219,220]. It is important to note that the bulk of the research on PGPF *Talaromyces* has been performed in soil; however, since researchers [180,181] observed *Talaromyces* persistence on roots in hydroponically grown crops, there is evidence that it can also act as a PGPF in hydroponics. 

*Talaromyces* species exhibit several mechanisms of biocontrol, either occupying root space to outcompete other microbes or producing hydrolytic enzymes such as chitinase or β-glucanase (enzymes used in the breakdown of fungal cell walls), giving them the potential to parasitize fungal pathogens. This is further supported by evidence that *T. pinophilus* formed pseudoappressorium with *B. cinerea* hyphae, resulting in disintegration of host cell walls and cytoplasmic disorganization [221].

Concomitantly, *Talaromyces* spp. both stimulate plant growth and induce disease resistance. In a study of *Capsicum annuum* L. infected with the fungal pathogen *Colletotrichum capsici*, *Talaromyces* sp. were capable of inhibiting *C. capsica* growth up to 89% [222]. In other studies, it was inferred that *Talaromyces* spp. are capable of parasitizing fungal pathogens through their production of hydrolytic enzymes [221,223]. Ref. [223] performed quantitative and qualitative studies on the capabilities of *T. pinophilus* to produce these enzymes while growing on media containing carboxymethylcellulose (CMC) and colloidal chitin. CMC and colloidal chitin media were selected because the metabolism of these substrates by the organism results in zones of clearance, indicating glucanase and chitinase activity, respectively [224,225]. These studies determined that *T. pinophilus* was a significant producer of glucanase, chitinase, and reactive oxygen species, supporting the idea of hyperparasitism of other fungal phytopathogens [223,226]

Concurrently, *Talaromyces* spp. can induce host defense mechanisms in plants, enhancing their resistance to pathogens. For instance, *T. wortmanni* secretes β-caryophyllene, a compound that stimulates plant growth and induces defense responses. β-caryophyllene adsorption on seedlings increased root length and reduced infection by the fungal pathogens *Colletotrichum higginsianum* and *B. cinerea* [220]. Additionally, β-caryophyllene was adsorbed onto seedlings of *Cucumis sativa*, *Nicotiana benthamiana*, and *Arabidopsis thaliana* without *T. wortmanni* present. β-caryophyllene adsorption resulted in increased resistance to *B. cinerea* and *C. orbiculare* along with enhanced growth. Interestingly, when inoculation of β-caryophyllene was performed less than 24 h prior to inoculation with a pathogen on 21-day-old plants, infection was not affected. These findings suggest that β-caryophyllene induces the plant’s host cell response to protect themselves from pathogens rather than directly inhibiting the pathogens [220]. This is further supported through findings that β-caryophyllene stimulated transcriptional activation of defense genes *npr-1*, *pad3,* and *pdf1.2* in *Arabidopsis*, indicating the activation of multiple defenses [220,227]. 

### 6.4. Trichoderma: Biocontrol and Immune Induction

*Trichoderma* is a genus of ascomycete filamentous fungi that is well-known for its use in biocontrol and plant growth promotion [228]. These fungi typically appear as white, cloudy tufts, which develop green pigmentation upon maturation. Currently, there are 441 known species of *Trichoderma* [229]. Several members employ methods of biocontrol, including occupational biocontrol, mycoparasitism, production of hydrolytic enzymes, and synthesis of antifungal metabolites. *Trichoderma* spp. tend to be fast-growing organisms, allowing them to occupy space and gather nutrients more effectively than pathogens [230]. *Trichoderma* spp. can reduce pathogenic infections both by leveraging their competitive fitness in a given ecological niche [231] and via mycoparasitism, i.e., direct parasitism of other fungi. For example, *T. harzianum* produces the hydrolytic enzyme chitinase to break down fungal cell walls. *Trichoderma* species also produce β-glucanases and proteases, which further contribute to dissolving pathogenic fungal cell walls [232,233]. 

The method by which *Trichoderma* spp. locate and parasitize prey follows a pathway of chemotaxis, adhesion and coiling around the host, appressorium formation, and penetration using mechanisms mentioned previously [182]. One study examined the effect of *T. virens* on *Rhizoctonia solani*, the causative agent of brown rot, concluding that *T. virens* has a significant antagonistic effect upon *R. solani* [234]. This antagonist effect was suggested to be the combined result of direct parasitism as well as volatile and non-volatile secretions. Another group reported that metabolites from *T. viride* significantly inhibit the wilt-specific form of *F. oxysporum* [235]. Indeed, *Trichoderma* strains can produce hundreds of antimicrobial substances such as harzianic acid, trichomycin, gelatinomycin, and chlorotrichomycin. These compounds inhibit various plant pathogens synergistically with cell wall-degrading enzymes and induce host cell responses [236,237]. 

*Trichoderma* spp. can also induce systemic resistance in plants, enhancing their ability to withstand pathogen attacks and abiotic stress. For instance, *Trichoderma* inoculation in chili pepper and tomato plants augmented the production of phenolic compounds and differentially expressed genes associated with stress responses and signaling [238]. Researchers examined *T. hamatum* modified gene expression of tomato plants inoculated with *Xanthomonas euvesicatoria*, a bacterial pathogen responsible for bacterial spot disease [239]. This study identified 45 differentially expressed genes, of which 14 were associated with abiotic/biotic stress reduction, indicating *T. hamatum* is involved in a physiological stress response [239,240]. *Trichoderma* spp. have also demonstrated an increase in resistance to *M. incognita*, a roundworm responsible for root knot disease [241]. Tomato plants inoculated with *M. incognita* and treated with *T. harzianum* experienced 62% control of *M. incognita*, as well as an upregulation of the genes *PAL*, *C4H*, *4CL*, *CAD*, *LPO*, *CCOMT*, *Tpx1*, and *G6PDH*. In a study performed by [242], inoculated tomatoes infected with the gray mold *B. cinerea* with *T. harzianum*. Plants inoculated with *T. harzianum* inhibited *B. cinerea* up to 62%, decreased reactive oxygen species, as well as increased expression of secondary metabolites, antioxidants, and the defense-related genes *C4H*, *CAD*, *CCOMT*, *G6PDH*, *PAL*, and *PR-1* [242]. These genes, along with the ones mentioned previously, are involved in stress response through support of lignification, secondary metabolism, and detoxification of reactive oxygen species [243]. The results from these studies demonstrate the ability of *Trichoderma* spp. to induce systemic resistance in plants. 

## 7. Plant Growth-Promotion by Phytohormone Production, Nutrient Acquisition, and Stress Regulation

### 7.1. Aspergillus: Phytohormone Production, Nutrient Acquisition, and Stress Regulation

*Aspergillus* species can also increase plant growth through processes that are independent of biocontrol and immune regulation in a variety of commercially important hydroponic crops, including cucumber, tomato, lettuce, kale, eggplant, and peppers [174,175]. Mechanisms of plant growth promotion include production of phytohormones, increased nutrient acquisition, and improved stress regulation (Figure 4). While the production of phytohormones such as ethylene and gibberellins by PGPF is well documented, the phytohormone most often produced by plant growth-promoting microorganisms, including *Aspergillus* species, is the auxin indole-3-acetic acid (IAA) [244]. IAA produced from either plants, bacteria, or fungi directs stem, root elongation, cell division, root, and vascular tissue differentiation [6]. For example, researchers studied the ability of *A. niger* to promote growth in seven different hydroponically grown crops, including lettuce, kale, eggplant, watermelon, melon, pepper, and tomato, where it increased shoot growth of all seven hydroponically grown crops relative to the uninoculated controls [174]. Growth increases ranged from 20% to 152% in melons and tomatoes, respectively. For lettuce and kale, 77% and 43% increases in shoot fresh weight also translate to significant increases in yield. In contrast, *A. niger* inoculation did not increase root growth in all seven crop types; inoculation increased root growth of hydroponic eggplant, tomato, pepper, and watermelon by 37%, 45%, 88%, and 72%, respectively, but did not significantly increase root growth in hydroponic lettuce, melon, or kale [174]. Therefore, this is an example of crop-specificity in plant-microbe interactions; the same fungus may promote growth in one crop and decrease growth or have no effect on another. The observed plant growth promotion in this study can be explained in part by IAA production, which is well-documented in *A. niger* [196,198,210]. Overall, plant growth promotion linked to IAA production has also been reported for a multitude of *Aspergillus* species, including but not limited to: *A. flavus*, *A. oryzae*, *A. tubingensis*, *A. alabamensis*, *A. fumigatus*, *A. chevalieri*, *A. egypticus*, *A. ustus*, *A. elegans*, *A. foetidus*, and *A. terreus* [175,186,197,200,203,210,245,246,247]. 

PGPF *Aspergillus* spp. are also capable of promoting plant growth by facilitating increased nutrient uptake by their host plant. The target nutrients, the mechanisms used, and the effect on plant growth can vary. The most common nutrients for PGPF *Aspergillus* spp. to facilitate plant uptake are iron and phosphate; however, nitrogen, ammonia, potassium, and zinc have also been reported targets [174,196,197,247]. 

Iron uptake is facilitated using low-molecular-weight protein complexes known as siderophores; siderophores bind and deliver iron to both the plant root cells and their associated fungus [6]. Inoculation with siderophore-producing *Aspergillus* species has been found to increase vegetative growth in tomato, eggplant, pepper, and legumes [196,200,203]. Iron is also required for chlorophyll synthesis [248]. Increased total chlorophyll has been documented in several crop types inoculated with siderophore-producing *Aspergillus* species [197,203,245]. 

Inoculation with phosphate-solubilizing *Aspergillus* species is reported to increase germination success, shoot and root length, fresh weight, number of leaves, chlorophyll content, and protein content. Improvements have been recorded in several hydroponic crops, including tomatoes, peppers, lettuce, kale, and eggplants [174,196,197,200,210,245]. It is worth noting that while common, high phosphate-solubilization is not found in every species of *Aspergillus*; only minimal phosphate-solubilization capacity by *A. foetidus* was recorded, while no phosphate-solubilization ability in *A. terreus* was noted [175,186]. 

Potassium solubilization, zinc solubilization, and nitrogen fixation are reported less frequently in *Aspergillus* species. A potassium-solubilizing strain of *A. niger* increased shoot height and fresh weight in hydroponic lettuce, kale, eggplant, watermelon, pepper, and tomato [174]. Recently, zinc-solubilizing *A. elegans* was reported to increase shoot and root length, chlorophyll content, and overall growth rate of cucumbers following inoculation [247]. Nitrogen fixation has been observed in *A. fumigatus* and was linked to increased germination success, vegetative growth, protein content, and chlorophyll content in the herb *Trigonella foenum-graecum* [197]. This observation is extremely novel, as fixation of atmospheric nitrogen by fungi has been proposed and disproven repeatedly in scientific history. Nitrogen fixation, the sequestration of atmospheric nitrogen gas (N_2_) into bioavailable ammonia (NH_3_), is not to be confused with the decomposition of organic nitrogen into ammonia. The former is done by bacteria, while the latter is performed by fungi [249]. The nitrogen fixation ability of this *A. fumigatus* strain should be confirmed biochemically and bioinformatically. 

Plant growth-promoting *Aspergillus* may also regulate plant stress. Plants produce ethylene, a phytohormone that can decrease growth during periods of biotic and abiotic stress. The last step in the pathway for ethylene biosynthesis in plants is the conversion of 1-aminocyclopropane-1-carboxylate (ACC) into ethylene by the enzyme ACC oxidase [250]. However, an alternate pathway for ACC biosynthesis exists in which the enzyme ACC deaminase metabolizes ACC into ammonia and α-ketobutyrate. Thus, increased activity of the ACC deaminase enzyme results in decreased ethylene production and a situation where plant growth is not inhibited despite the presence of stressors [251]. ACC deaminase produced by PGPF, rather than by the plant, can have the same effect. High levels of ACC deaminase activity were found in a strain of *A. niger*; inoculation of this fungus onto *Phaseolus vulgaris* increased plant vegetative growth parameters, including shoot and root length, and total fresh weight [196]. Related research has described ACC deaminase activity in *Aspergillus* species, including *A. fumigatus*, *A. awamori*, and *A. aculeatus*, and their positive effects on corn and rice production [252,253,254]. Unfortunately, the role of ACC deaminase produced by *Aspergillus* in hydroponic crop production remains to be elaborated.

### 7.2. Penicillium: Phytohormone Production, Nutrient Acquisition, and Stress Regulation

As with *Aspergillus* PGPF, the exact mechanism of plant growth-promotion by *Penicillium* varies with fungal species and crop type. Broadly, the mechanisms of *Penicillium* plant growth-promotion fall under the categories of phytohormone production and increased nutrient acquisition, as depicted in Figure 4. The production of the phytohormones IAA, gibberellin, and ABA by *Penicillium* spp. has been linked to plant growth-promotion in a variety of hydroponic crops, including tomatoes, lettuce, cucumber, pepper, and eggplant [175,210,214,216,255,256,257]. As with other PGPF genera, IAA was the most frequently reported phytohormone produced by *Penicillium*. For example, inoculation of tomatoes with IAA-producing *P. oxalicum* increased shoot length, root length, and the number of leaves with respect to uninoculated controls [198]. In cucumbers, *P. menonorum* inoculation resulted in increased yields and biomass [255]. In Reda F1 hybrid peppers, inoculation with an IAA-producing consortium containing *Trichoderma harzianum* and *P. expansum* conferred increases in shoot length, root length, and the number of leaves [216]. GA-production by *P. resedanum* was linked to increased shoot length, biomass, photosynthetic rate, stomatal conductance, chlorophyll content, and leaf area, despite the imposition of heat, salinity, and temperature stress on pepper plants [257]. 

Increased phosphorous uptake has been repeatedly linked to increases in a variety of vegetative growth parameters in plants [258]. Therefore, it is no surprise that phosphate-solubilizing *Penicillium* species have been linked with increased plant growth on numerous occasions. The ability to solubilize phosphates was reported in *P. pinophilum*, *P. oxicalum*, *P. menonorum*, *P. expansum*, *P. allahabense*, and two uncharacterized *Penicillium* spp. [175,198,210,216,255,259]. Plant growth-promotion by *Penicillium* that can be at least partially attributed to increased nutrient acquisition has been observed in tomato, lettuce, cucumber, and pepper. Specifically, increased uptake of phosphorus and iron evidently are the primary nutrients increased by PGPF *Penicillium* [216,255,257,259]. For example, inoculation of 400 tomato seeds with *Penicillium* strain RFUOM14 resulted in significant increases in germination success and seedling vigor compared to the uninoculated controls [259]. In another study, the phosphate-solubilization capacity of *P. oxicalum* was linked to increased shoot length, root length, and the number of leaves in tomatoes [198]. Ref. [255] quantified a 14% increase in phosphorus content of cucumber plants following their inoculation with *P. menonorum*; the inoculated plants were 53% larger than uninoculated controls, with 57% greater root mass and 52% greater shoot mass [255]. 

*Penicillium* also increases plant growth by making iron more bioavailable to hydroponic crops. Recollect that iron is involved in chlorophyll biosynthesis and energy-producing redox reactions in plants, and microorganisms that secrete siderophores can increase iron uptake by host plants [6,248]. Siderophore production has been observed in *P. pinophilum*, *P. oxalicum*, *P. menonorum*, *P. expansum*, and *P. allahabense* [175,198,216,255,260]. Inoculation with siderophore-producing *Penicillium* species is frequently associated with increases in photosynthetic pigment content, which explains associated increases in photosynthetic rate, vegetative growth, and yield. For example, *P. pinophilum* inoculation of both tomatoes and lettuce increased chlorophyll content, allowing subsequent increases in biomass and yield [258,260]. Inoculation of tomatoes with *P. oxalicum* increased total photosynthetic pigment content, shoot length, root length, and the number of leaves [198]. Cucumbers inoculated with *P. menonorum* exhibited increased chlorophyll content compared to uninoculated controls [255]. 

### 7.3. Talaromyces: Phytohormone Production, Nutrient Acquisition, and Stress Regulation

*Talaromyces* also increase plant growth via phytohormone production and improving nutrient acquisition. Several species of *Talaromyces* have been found to synthesize IAA [223,261,262,263,264]. *T. pinophilus* can produce IAA through both trp-dependent and independent pathways. Additionally, *T. pinophilus* exhibits substantial phosphate solubilization capabilities. This ability was assessed qualitatively through growth on Pikovskaya’s agar and quantitatively through growth in Pikovskaya’s liquid media stained with stannous chloride [222]. *T. pinophilus* is also capable of producing iron-chelating siderophores [223,265]. On another note, *T. omanensis* improves drought-stressed tomato plants’ reproductive and physiological traits. A higher concentration of gibberellic acid (GA3) in treated plants suggests that *T. omanensis* may enhance drought tolerance through hormonal modulation [219]. More studies should be conducted on *Talaromyces* to explore if the array of phytohormone production and nutrient acquisition strategies matches the diversity of other PGPF.

### 7.4. Trichoderma: Phytohormone Production, Nutrient Acquisition, and Stress Regulation

*Trichoderma* spp. synthesize various phytohormones, which are integral to their role in promoting plant growth (Figure 4) [266,267]. This latter study quantified the IAA and gibberellin production of 15 *Trichoderma* isolates. All 15 isolates were found to produce IAA and gibberellin in various concentrations, with isolate T11 producing the highest IAA concentration at 71.2 mg/L and T3 producing the highest gibberellin concentration at 4.6 mg/L. Furthermore, various studies have concluded that *Trichoderma* strains can produce ACC deaminase, aiding in plant growth under stressful conditions [268]. In hydroponic systems, these fungal endophytes can produce siderophores, phosphatases, phytases, and organic acids [183,269,270] as well as being linked to an increase in nutrient uptake in plants [271]. One study examined the effect *T. harzianum* has upon hydroponically grown tomatoes in nutrient-limiting conditions [183]. Tomato plants were grown in hydroponic systems individually with the nutrients phosphorus, potassium, iron, copper, zinc, and manganese. An increased growth response was correlated with significant increases in the concentrations of copper, iron, sodium, and zinc in inoculated roots. Another study [272] examined the growth of tomato plants in reduced nitrogen, phosphorus, and potassium (N-P-K) conditions. The results from this experiment revealed that when supplemented with *Trichoderma*, tomato plants had a growth increase of 101% compared to a half dose of N-P-K fertilizer and 11% compared to the standard N-P-K dose. Additionally, tomato fruits were found to have higher levels of these nutrients and key biomolecules such as lycopene and β-carotene. Indeed, *Trichoderma* spp. have also been linked to stress reduction from environmental conditions [273]. *Trichoderma* inoculation in lettuce irrigated with arsenic-containing water resulted in significant growth promotion and reduced arsenic levels in the plants, indicating enhanced abiotic stress tolerance [274]. This has implications for the utilization of *Trichoderma* for growth under suboptimal growth conditions or water quality. 

## 8. Arbuscular Mycorrhizal Fungi (AMF)

AMF are a group of mycorrhizal fungi characterized by the formation of structures known as arbuscules within plant cells. Unlike ectomycorrhizae, which form structures between root cells, AMF arbuscules develop within the cortical cells of roots [275]. These arbuscules serve as sites for bidirectional nutrient transfer, where the fungus provides essential nutrients from the environment to the plant in exchange for photosynthetic products. AMF can form associations with an estimated 200,000 different plant species, and up to 20% of their photosynthetic products will be transferred to the fungus [276]. Common AMF genera used to promote plant growth include *Rhizophagus*, *Glomus*, *Claroideoglomus*, *Gigaspora*, and *Funneliformis* [277]. These fungi enhance plant growth by forming extensive hyphal networks that acquire and transport water and nutrients to the plant host [256,278,279]. Ref. [280] studied the effects of AMF on plant growth in nutrient-limiting conditions and found that tomato plants inoculated with AMF and supplemented with 50% of the standard fertilizer amount produced growth results comparable to those plants receiving 100% of the standard fertilizer. Another study [281] examined the impact of AMF on cherry tomato plants grown in a hydroponic system under both nutrient-limiting and non-limiting conditions. These results showed that AMF inoculation increased fruit weight by 64% and had higher overall nutrient concentrations compared to the control. 

## 9. Discussion

Plants have coevolved with microbes for hundreds of millions of years, leading to dynamic and complex relationships between the microbes and plants [171]. These interactions are stable and are classified as either commensal, mutualistic, or parasitic [282]. This categorization offers a useful conceptualization to study host–microbe relationships, but it is a severe oversimplification. Mainly because (i) plant–microbe interactions are rarely limited to two partners [283] and (ii) the observed interactions are not continuous and can vary based on the host and the changing environmental conditions [282]. This is similar to the accuracy of fungal pathogenic lifestyle classification [49]. Indeed, most fungal and oomycete pathogens actively switch between biotrophic and necrotrophic phases, depending on the immune response of the plant as well as the environmental conditions, which can include other microbes. This is illustrated by *F. oxysporum* strains that are regarded as host specific because they can only cause disease in a narrow subset of plants, despite asymptomatically colonizing others. In some cases, these “cryptic” asymptomatic infections can cause host cell death [284]. In other cases, the presence of certain microbes in the plant can change the behavior of surrounding microbes [132]. Thus, root exudates from plants colonized with non-pathogenic *F. oxysporum* reduce the rate of germination of other pathogenic *F. oxysporum* strains. Despite the benefits of having a conceptualized reductionist view, it is no longer possible to ignore the importance of the complex plant microbiome due to its capacity to control disease or contribute to plant health promotion [285]. 

Further exploration of these complex interactions can lead to better practices preserving plant health, especially in hydroponics. Additional studies on plant growth promoting fungi are tabulated in Supplemental Table S1 and Table S2 [176,286,287,288,289,290,291,292,293,294,295,296,297,298,299,300,301,302,303,304,305,306,307,308,309,310,311,312,313,314,315,316,317,318,319]. Indeed, soilless cultures can be the perfect environment for studying and tapping into the potential of the microbiome, given that the plants constitute a major source of organic matter and secondary metabolites [319], shaping the established microbiome [320], and influencing plant health in a dynamic reciprocal relationship. For instance, a recent study [321] recorded a positive association between *Flavobacterium* and *Pseudomonas* and the severity of *Phytophtora* symptoms in hydroponically grown lettuce plants. This suggests that the composition of the bacterial microbiome can influence the probability for disease development for fungi and oomycetes, or at the very least act as a biomarker for disease risk. Furthermore, both host tryptophan metabolism and bacterial commensals are correlated with shifts in the mycobiome and subsequent impairment of plant growth [322]. Thus, changes in the mycobiome can also affect plant health in a disease-independent manner and should not be ignored. 

There is currently a shift in research, given the increasing importance of hydroponics as a sustainable agricultural model. There are, however, a few limitations that need to be addressed. First is the difficulty of fungal and oomycetes genomics [323]. The lack of proper tools for elucidating both the present fungal and oomycetes population, as well as their genes, is an important obstacle that needs to be bridged. Second is the reciprocal nature of microbial interactions. As stated previously, most studies delving into disease development or PGPF interactions are carried out in a reductionist design, i.e., just because a single fungus can inhibit the growth of a phytopathogen or increase plant health in an experimental setting, it does not guarantee the same outcome in a commercial growing operation. Understanding and then optimizing the mycobiome should help to increase crop yields in soilless cultures. Studies are encouraged to scale up to larger hydroponic systems to ensure their conclusions on which PGPF increase yields translate well to commercial systems. Including robust data on whether an inoculant contributes to an increase in marketable yield (both counts and total mass) is very informative to growers, more so than changes in shoot mass or dry weights. Additionally, researchers are encouraged to explore PGPF that have less data in hydroponic systems, such as *Talaromyces* and AMF. These organisms have the potential to greatly increase crop yields in the food supply chain and are worth exploring in greater detail.

## 10. Concluding Remarks

Transitioning to hydroponic cultures is increasingly seen as a viable solution to the emerging climate and population challenges. A pivotal principle of hydroponics is homogeneity and consistency. Growers seek to maintain uniform and optimal conditions to optimize plant growth. Yet, this homogeneity may also provide optimal conditions for pathogen growth, especially for fungi and oomycetes that are able to persist for long periods of time in water. Consequently, fungal phytopathogens constitute a significant threat to hydroponic operations. However, multiple fungal genera exhibit biocontrol and PGPF capacity. These include but are not limited to, direct pathogen inhibition, hyperparasitism, nutrient uptake, and phytohormone stimulation. Indeed, fungal–plant interactions in hydroponics constitute a promising avenue of future high-impact research. Research that determines effective new methods to control fungal phytopathogens and formulates robust fungal inoculants to increase crop yields has the potential to revolutionize modern agriculture and help to feed the world’s growing population. Researchers are encouraged to explore the hydroponics microbiome in all of its facets: the good, the bad, and the fungal.

## Data Availability

Not applicable.

## Supplementary Materials

The following supporting information can be downloaded at: https://www.mdpi.com/article/10.3390/biology13121014/s1, Table S1: Summary of PGPF as biocontrol agents against phytopathogens of hydroponic crops; Table S2: Summary of direct mechanisms of plant growth-promotion by PGPF.
