# Supplementary material for: The Good, the Bad, and the Fungus: Insights into the Relationship Between Plants, Fungi, and Oomycetes in Hydroponics
Source: Biology (Basel). 2024 Dec 4;13(12):1014. doi: 10.3390/biology13121014 (PMC11673877; doi:10.3390/biology13121014)
Supplement: Supplementary file 1 [file biology-13-01014-s001.zip › biology-3333299- supplementary Table S1.pdf]

Supplemental Table S1. Summary of PGPF as biocontrol agents against phytopathogens of hydroponic crops

| **Species** | **Affected crop** | **Pathogen** | **Biocontrol Mechanism** | **Reference** |
|---|---|---|---|---|
| ***Aspergillus*** | | | | |
| *A. ustus* | *Arabidopsis thaliana* | *Botrytis cinerea*<br>*Pseudomonas syringae pv. tomato DC3000* | ISR via SA-, JA-, and ethylene-mediated pathways<br>Regulation of camalexin defense and oxidative-burst related genes | [246] |
| *A. fumigatus* | Tomato | *Fusarium oxysporum* | Antibiosis | [195] |
| *A. niger, A. flavus* | Tomato | *F. oxysporum* | ISR | [198] |
| *A. niger, A. flavus* | Tomato | *F. oxysporum* | Competition (siderophore production)<br>Antibiosis (HCN production) | [210] |
| *A. fumigatus, A. flavus, A. nidulans* | Tomato | *F. oxysporum* | ISR<br>Occupational biocontrol | [194] |

| | | | | |
|---|---|---|---|---|
| *A. oryzae* | Eggplant | *Meloidogyne incognita* | Competition (siderophore production)<br><br>Antibiosis (toxic VOC production, HCN production) | [203] |
| *A. tubingensis, A. alabamensis, A. oryzae* | Pepper | *F. oxysporum* | Competition (siderophore production)<br><br>Antibiosis (HCN production)<br><br>ISR | [200] |
| *A. chevalieri, A. egypticus* | Broad bean | *Alternaria solani* | Competition (siderophore production)<br><br>Antibiosis (antagonistic metabolite production) | [245] |
| *A. terreus* | Tomato | *P. syringae pv. tomato DC3000* | ISR via SA-mediated signalling | [186] |
| ***Penicillium*** | | | | |
| sp. | Tomato | *F. oxysporum* | Competition (siderophore production)<br><br>Antibiosis (HCN production) | [210] |

| | | | | |
|---|---|---|---|---|
| *P. viridicatum* | *A. thaliana* | P. syringae pv. tomato DC3000 | ISR via ethylene-mediated signalling | [211] |
| *P. viridicatum* | Cucumber | *Rhizoctonia solani, Colletotrichum orbiculare* | ISR | [215] |
| *P. chrysogenum* | Cucumber | *M. incognita* | ISR involving upregulation of SA- and JA-regulated defense genes | [208,209] |
| *P. simplicissimum* | *A. thaliana* | *P. syringae pv. tomato DC3000* | ISR involving upregulation of MYB44 | [177] |
| *P. simplicissimum* | Cucumber | *Mycus persicae* | ISR via SA- and JA-mediated pathways | [207] |
| *P. pinophilum* | Tomato | Verticillium dahliae | Occupational biocontrol<br>Antibiosis (production of antagonistic compounds)<br>ISR | [260] |
| *P. oxicalum* | Tomato | *F. oxysporum* | Antibiosis (production of antifungal compounds, including HCN)<br>ISR | [198] |

| | | | | |
|---|---|---|---|---|
| *P. expansum* | Pepper | *F. oxysporum* | Antibiosis (production of antifungal compounds, including HCN)<br><br>Competition (siderophore production) | [216] |
| *P. commune* | Eggplant | *Sclerotinia scleotiorum* | Antibiosis (production of antifungal compounds) | [214] |
| *P. rubens* | Tomato | *F. oxysporum f. sp. lycopersici* | ISR involving upregulation of the xylanolytic system | [176] |
| *P. rubens* | Tomato | *F. oxysporum f. sp. lycopersici* | ISR involving production of secondary metabolites<br><br>Lytic enzyme production | [178] |
| ***Fusarium*** | | | | |
| *F. equiseti* | Peas | *Fusarium avenaceum, Peyronellaea pinodella* | Occupational biocontrol | [286] |
| *F. equiseti* | Tomato | *P. syringae pv. tomato DC3000* | Occupational biocontrol<br><br>ISR via SA-mediated signalling | [287] |

| | | | | |
|---|---|---|---|---|
| *F. oxysporum* | Cucumber | *F. oxysporum f. sp. cucumerinum* | ISR via SA-, JA-, and ethylene-signalling | [288] |
| *F. oxysporum* | Tomato | *F. oxysporum f. sp. lycopersici* | ISR via production of cysteine-rich proteins | [289] |
| *F. oxysporum* | Lettuce | *F. oxysporum f. sp. lactucae, Curvularia sp., F. semitectum, Rhizoctonia spp.* | Occupational biocontrol<br>Antibiosis | [290] |
| *F. oxysporum Fo47* | Tomato | *F. oxysporum f. sp. lycopersici* | Occupational biocontrol<br>ISR | [291] |
| *F. solani K* | Tomato | *Two-spotted spider mite (Tetranychus solani)* | ISR | [292] |
| ***Phoma*** | | | | |
| sp.GS8-1 | Cucumber | *C. orbiculare* | Lytic enzyme production (endochitinase, endoglucanase)<br>ISR | [293] |

| | | | | |
|---|---|---|---|---|
| sp. GS8-2 | *A. thaliana, cucumber* | Cucumber mosaic virus (CMV) | ISR | [294] |
| sp. GS8-3 | Cucumber | Cucumber mosaic virus (CMV) | ISR involving upregulation of defense- and heat shock-related proteins) | [295] |
| sp. GS8-3 | Pepper | *R. solani, F. oxysporum f. sp. capsici* | ISR involving upregulation of CaPR4 | [296] |
| spp. GS8-1, GS8-2, GS8-3 | Cucumber | *C. orbiculare* | ISR involving upregulation of CaPR4 | [297] |
| spp. GS10-1, GS14-1 | Tomato | *P. syringae pv. tomato DC3000* | Occupational biocontrol<br><br>ISR via SA-mediated signalling | [287] |
| ***Trichoderma*** | | | | |
| *T. asperellum T34* | Tomato | *F. oxysporum f.sp. lycopersici* | | [298] |
| *T. asperellum Hu1* | Tomato | *Alternaria alternata* | ISR via SA- and JA-mediated pathways<br><br>Occupational biocontrol | [299] |

| | | | | |
|---|---|---|---|---|
| *T. asperellum* | Tomato | *F. oxysporum, B. cinerea* | ISR | [300] |
| *T. asperellum* | Eggplant | *S. sclerotiorum* | Antibiosis (production of antifungal compounds)<br>ISR | [214] |
| *T. asperellum NST-009* | Lettuce | *Cercospora lactucae-sativae* | Occupational biocontrol<br>Competition for nutrients<br>Mycoparasitism<br>Antibiosis (production of antifungal compounds) | [301] |
| *T. harzianum* | Peas | *F. oxysporum* | Antibiosis (production of antibiotic and antifungal compounds)<br>Lytic enzyme production (proteases, chitinases, ligninase, cellulase)<br>ISR | [302] |
