# Supplementary material for: The Good, the Bad, and the Fungus: Insights into the Relationship Between Plants, Fungi, and Oomycetes in Hydroponics
Source: Biology (Basel). 2024 Dec 4;13(12):1014. doi: 10.3390/biology13121014 (PMC11673877; doi:10.3390/biology13121014)
Supplement: Supplementary file 1 [file biology-13-01014-s001.zip › biology-3333299- supplementary Table S2.pdf]

Supplemental Table S2. Summary of direct mechanisms of plant growth-promotion by PGPF

| Species | Affected crop | Result | Mechanism | Reference |
|---|---|---|---|---|
| ***Aspergillus*** | | | | |
| spp. PPA1 | Cucumber | Increased root and shoot growth, and chlorophyll content | | [309] |
| *A. ustus* | *Arabidopsis thaliana* | Increased root and shoot growth | Phytohormone production (IAA, GAs) | [246] |
| *A. fumigatus* | Fenugreek | Increased germination success, root and shoot growth, chlorophyll content, and total protein content | Phytohormone production (IAA)<br><br>Nutrient acquisition (siderophore production, phosphate solubilization, nitrogen fixation) | [197] |
| *A. fumigatus* | Tomato | Increased shoot growth, protein content, and chlorophyll content | Stress regulation (antioxidant production) | [195] |
| *A. fumigatus, A. flavus, A. nidulans* | Tomato | Increased carbohydrate content, protein content, photosynthetic pigment content | Stress regulation (antioxidant production) | [194] |
| *A. niger* | Lettuce, kale, eggplant, | Increased root and shoot growth | Phytohormone production (IAA, GAs) | [174] |

| | | | | |
|---|---|---|---|---|
| | watermelon, melon, pepper, tomato | | Nutrient acquisition (phosphate and potassium solubilization) | |
| *A. niger* | Common bean | Increased root and shoot growth | Phytohormone production (IAA)<br>Nutrient acquisition (siderophore production, phosphate solubilization)<br>Stress regulation (ACC deaminase) | [196] |
| *A. niger, A. flavus* | Tomato | Increased root and shoot growth | Phytohormone production (IAA)<br>Nutrient acquisition (siderophore production, phosphate solubilization) | [198] |
| *A. niger, A. flavus* | Tomato | Increased carbohydrate content, protein content<br>Decreased biomarkers of oxidative stress | Phytohormone production (IAA, ABA)<br>Nutrient acquisition (siderophore production, phosphate solubilization) | [210] |

| | | | | |
|---|---|---|---|---|
| | | | Stress regulation (antioxidant production) | |
| *A. tubingensis, A. alabamensis, A. oryzae* | Pepper | Increased root and shoot growth, photosynthetic pigment content | Phytohormone production (IAA)<br><br>Nutrient acquisition (siderophore production) | [200] |
| *A. foetidus* | Cucumber | Increased root and shoot growth | Phytohormone production (IAA) | [175] |
| *A. oryzae* | Eggplant | Increased chlorophyll content | Phytohormone production (IAA)<br><br>Nutrient acquisition (siderophore production) | [203] |
| *A. elegans* | Cucumber | Increased root and shoot growth, and chlorophyll content | Phytohormone production (IAA)<br><br>Nutrient acquisition (zinc solubilization) | [247] |
| *A. chevalieri, A. egypticus* | Broad bean | Increased shoot and root growth, chlorophyll content, yield<br><br>Decreased biomarkers of oxidative stress | Phytohormone production (IAA)<br><br>Nutrient acquisition (siderophore production, phosphate solubilization) | [245] |

| | | | | |
|---|---|---|---|---|
| | | | Stress regulation (antioxidant production) | |
| *A. terreus* | Tomato | Increased shoot growth | Phytohormone production (IAA) | [186] |
| ***Penicillium*** | | | | |
| sp. | Tomato | Increased carbohydrate content, protein content<br>Decreased biomarkers of oxidative stress | Phytohormone production (IAA, ABA)<br>Nutrient acquisition (siderophore production, phosphate solubilization)<br>Stress regulation (antioxidant production) | [210] |
| sp. RFUOM14 | Tomato | Increased germination success and seedling vigor | Nutrient acquisition (phosphate solubilization) | [259] |
| *P. viridicatum* | *A. thaliana* | Increased shoot growth | | [211] |
| *P. viridicatum* | Cucumber | Increased root and shoot growth | | [215] |
| *P. chrysogenum* | Cucumber | Increased germination, root and shoot growth | | [209] |

| | | | | |
|---|---|---|---|---|
| *P. pinophilum* | Tomato | Increased yield, chlorophyll content, nutrient content, biomass, and root growth | Nutrient acquisition (siderophore production, phosphate solubilization)<br><br>Stress regulation (antioxidant production) | [260] |
| *P. pinophilum* | Tomato, lettuce | Increased yield, shoot and root growth, nutrient content, chlorophyll content | Phytohormone production (IAA, GA)<br><br>Nutrient acquisition (siderophore production, phosphate solubilization) | [256] |
| *P. oxicalum* | Tomato | Increased root and shoot growth, carbohydrate content, chlorophyll content | Phytohormone production (IAA)<br><br>Nutrient acquisition (siderophore production, phosphate solubilization)<br><br>Stress regulation (antioxidant production) | [198] |
| *P. menonorum* | Cucumber | Increased yield, root and shoot growth, chlorophyll content, starch content, protein content, nutrient content | Phytohormone production (IAA)<br><br>Nutrient acquisition (siderophore production, phosphate solubilization) | [255] |

| | | | | |
|---|---|---|---|---|
| | | | Phytohormone production (IAA) | |
| *P. expansum* | Pepper | Increased root and shoot growth, protein content, carbohydrate content, chlorophyll content | Nutrient acquisition (siderophore production, phosphate solubilization)<br>Stress regulation (antioxidant production) | [216] |
| *P. commune* | Eggplant | Increased yield, root and shoot growth | Phytohormone production (IAA, GAs) | [214] |
| *P. resedanum LK6* | Pepper | Increased root and shoot growth under salinity, drought, and temperature stress conditions | Phytohormone production (GA)<br>Stress regulation (regulation of ABA-mediated stress response, SA-mediated systemic resistance) | [257] |
| *P. allahabense* | Cucumber | Increased root and shoot growth | Phytohormone production (IAA, GA)<br>Nutrient acquisition (siderophore production, phosphate solubilization) | [175] |
| ***Fusarium*** | | | | |

| | | | | |
|---|---|---|---|---|
| sp. PPF1 | Indian spinach | Increased germination success, seedling vigor, root and shoot growth, and chlorophyll content | | [310] |
| *F. equiseti* | Peas | Increased root and shoot growth | | [286] |
| *F. equisetii GF18-3, GF19-1* | Tomato | Increased root and shoot growth | | [287] |
| *F. oxysporum* | *A. thaliana* | Increased root and shoot growth and chlorophyll content | Phytohormone regulation (auxin signalling mediated by fungal volatiles) | [311] |
| *F. oxysporum FO12* | Cucumber | Increased root and shoot growth, number of flowers, | Nutrient acquisition (ferric reductase activity, upregulation of iron-deficiency genes, rhizosphere acidification)<br><br>Phytohormone regulation (upregulation of ethylene biosynthetic genes) | [312] |
| *F. oxysporum FO12* | Eggplant, cucumber | Increased root and shoot growth | | [313] |
| *F. oxysporum FO12* | Pepper | Increased root growth | | [313] |
| *F. oxysporum F221-B* | Lettuce | Increased germination success, rate and seedling length, yield and root growth, and chlorophyll content | | [290] |

| | | | | |
|---|---|---|---|---|
| ***Phoma*** | | | | |
| sp. GS8-2 | *A. thaliana* | Increased shoot growth and total biomass | | [294] |
| sp. GS8-2 | Cucumber | Increased yield and shoot growth | | [294] |
| sp. GS8-3 | Cucumber | Increased yield and shoot growth | Phytohormone production | [295] |
| sp. GS8-3 | Pepper | Increased shoot growth | Stress regulation (antioxidant production) | [296] |
| spp. GS10-1, GS14-1 | Tomato | Increased root and shoot growth | | [294] |
| ***Trichoderma*** | | | | |
| *T. harzianum SQR-T037* | Tomato | Increased root and shoot growth under copper-deficient conditions | Nutrient acquisition (mineral solubilization, organic acid production, ferric reductase activity, siderophore production) | [271] |
| *T. harzianum* | Lettuce | Increased yield, root growth, and nutrient content | Nutrient acquisition | [314] |
| *T. harzianum* | Pepper | Increased root and shoot growth, chlorophyll content, protein content, and carbohydrate content | Stress regulation (antioxidant production) | [216] |

| | | | | |
|---|---|---|---|---|
| | | | Phytohormone production (IAA) | |
| *T. harzianum* | Peas | Increased yield, seed quality, shoot and root growth, chlorophyll and carotenoid content | Nutrient acquisition (phosphate solubilization, siderophore production) | [302] |
| | | | Stress regulation (antioxidant production) | |
| *T. harzianum, T. brevicompactum, T. gamsii* | Tomato | Increased root and shoot growth, and chlorophyll content | Phytohormone production (IAA)<br><br>Nutrient acquisition (phosphate solubilization) | [305] |
| *T. harzianum T-203* | Cucumber | Increased seedling emergence, root and shoot growth, chlorophyll content, and nutrient content | Phytohormone production<br><br>Nutrient acquisition (mineral solubilization) | [184] |
| *T. harzianum, T. viride* | Tomato | Increased root and shoot growth | Stress regulation (antioxidant production) | [304] |
| T. harzianum T22, T. atroviride P1 | Lettuce | Increased total biomass under arsenic-toxicity conditions | Stress regulation | [274] |

| | | | | |
|---|---|---|---|---|
| *T. asperellum* | Eggplant | Increased root and shoot growth | | [214] |
| *T. asperellum T34* | Tomato | Increased root and shoot growth, and photosynthetic rate under iron-toxicity conditions | Nutrient sequestration (siderophore production) | [298] |
| *T. asperellum Hu1* | Tomato | Increased shoot growth, protein content, carbohydrate content, and nitrogen uptake | Stress regulation (antioxidant production) | [299] |
| *T. asperellum T34* | Cucumber | Increased nutrient content | Nutrient acquisition | [315] |
| *T. asperellum NST-009* | Lettuce | Increased yield, root growth, and nutrient content | Nutrient acquisition (phosphate solubilization)<br><br>Stress regulation (antioxidant production) | [301] |
| *T. asperellum* | Tomato | Increased root and shoot growth | Stress regulation (antioxidant production) | [300] |
| T. afroharzianum TM2-4 | Tomato | Increased germination, seedling vigor, shoot growth, and root activity | Phytohormone regulation<br><br>Stress regulation (antioxidant production) | [316] |

| | | | | |
|---|---|---|---|---|
| *T. atroviride* | *A. thaliana* | Increased total biomass and altered root network architecture | Phytohormone production (IAA, ethylene)<br><br>Phytohormone regulation involving MPK6 activity | [317] |
| ***Talaromyces*** | | | | |
| sp. | Chili pepper | Increased germination success, seedling vigor, shoot growth, chlorophyll content, yield, and flowering rate | Phytohormone production (IAA)<br><br>Nutrient acquisition (siderophore production, phosphate solubilization) | [222] |
| *T. omanensis* | Tomato | Increased yield, chlorophyll and carotenoid content, root and shoot growth, and number of flowers<br><br>Did not significantly alter flavour parameters (Brix, titratable acidity) | Phytohormone production (GA)<br><br>Stress regulation (antioxidant production) | [219] |
| *T. variabilis* | Cucumber, tomato | Increased seedling survival | Nutrient acquisition (siderophore production) | [308] |

| | | | | |
|---|---|---|---|---|
| *T. flavus var. flavus CBS 310.38* | Lettuce | Increased shoot growth | Nutrient acquisition (zinc sequestration) | [318] |
